# The oral und systemic health risks of sugar consumption: an umbrella review

**DOI:** 10.1007/s00784-026-07191-1

**Published:** 2026-09-30

**Authors:** J. P. Woelber, N. Wirth, A. Dallmer-Zerbe, C. Tennert

**Affiliations:** 1 Department of Operative Dentistry, Periodontology, and Pediatric Dentistry, Medical Faculty Carl Gustav Carus, Technische Universität Dresden, Dresden, Germany; 2 Private Practice, Solothurn, Switzerland; 3 Private Practice, Langenfeld, Germany; 4 Department of Restorative, Preventive and Pediatric Dentistry, School of Dental Medicine, University of Bern, Freiburgstrasse 7, Bern, CH-3010 Switzerland

**Keywords:** Sugar, Sugar-sweetened beverages, Artificially sweetened beverages, Oral health, Obesity, Cardiometabolic risk factors

## Abstract

**Objective:**

The recommendation of free sugar reduction in dental practice is mostly given with regard to caries prevention but has also further health impacts. This umbrella review aimed to comprehensively display the shared health consequences of sugar consumption in both oral and general health.

**Materials and methods:**

Within an umbrella review, two independent reviewers identified and analysed systematic reviews examining free sugar consumption alone or as a part of dietary patterns and its association to described diseases in three databases (Epistemonikos, Pubmed, Cochrane Library). Electronic databases were searched up to March 2026, findings were synthesised narratively. Risk of bias was assessed using AMSTAR-2.

**Results:**

The literature search identified 10,593 articles, of which 633 were further assessed for eligibility. Finally, 228 articles were included after full-text analysis. Results showed that sugar intake was consistently associated with increased risks of 18 generalised negative health outcomes. Strongest evidence was found for type 2 diabetes, obesity, cardiovascular and all-cause mortality, oral diseases, irritable bowel syndrome, and cognitive disorders. Furthermore, a positive association was found between excessive sugar consumption and cancer, non-alcoholic fatty liver disease, kidney disease, gout, asthma, poor sleep, reduced bone density, mood disorders, telomere shortening, and fertility impairments. AMSTAR-2 showed that most reviews had low or critically low methodological quality, while about one third demonstrated moderate to high confidence. Most studies (68%) investigated sugar in form of sugar-sweetened beverages.

**Conclusions:**

Within the limitations of this umbrella review, available evidence indicates that avoiding free sugar intake contributes to both reduced chronic disease burden and improved oral health. Against this background, avoiding free sugar, as recommended by dentists, is a key shared health factor that should be emphasized in the context of caries, periodontal disease, and in terms of general primary prevention.

**Clinical relevance:**

Dental advised and assisted sugar reduction is an important preventive measure not only for oral health but also for several general health outcomes. After synthesizing and evaluating the available systematic reviews, this study highlights the crucial need to reduce sugar consumption as an important factor in improving both systemic and oral health.

**Systematic review registration:**

The systematic review was registered in the international register for systematic reviews Prospero. Systematic review registration CRD42023380458.

**Supplementary Information:**

The online version contains supplementary material available at https://doi.org/10.1007/s00784-026-07191-1.

## Introduction

Excessive sugar consumption has become a significant public health issue since the start of industrial sugar production. A growing body of evidence links high sugar intake (particularly sugar-sweetened beverages (SSB)) to obesity, type 2 diabetes mellitus (T2DM), cardiovascular diseases, non-alcoholic fatty liver disease (NAFLD), and several oral health conditions, such as dental caries [1–7] and periodontitis [8–10]. These associations are explained by multifactorial mechanisms which include chronic inflammation, oxidative stress, total calories overload, insulin resistance, disproportionate satiety, changes in oral and gut microbiota and bacterial metabolism/metabolites [11, 12]. The World Health Organization (WHO) estimates that dental caries affects approximately 60–90% of school-aged children and most adults worldwide [4]. Meanwhile, periodontal diseases, with a global prevalence of around 50%, are a leading cause of tooth loss and reduced quality of life and are increasingly associated with multiple systemic conditions [8]. Periodontal disease affects approximately 3.9 billion people worldwide and ranks as the 11th most prevalent disease [10].

Dental caries is defined as the destruction of tooth enamel caused by acids produced by cariogenic bacteria during the fermentation of sugars and starches [13]. Caries develops through the interaction of bacteria, fermentable carbohydrates, and susceptible tooth surfaces and progresses when demineralization exceeds the protective effects of saliva and remineralization. High and frequent consumption of free sugars promotes acid production by oral bacteria, leading to enamel demineralisation and an increased risk of dental caries. According to the World Health Organization, free sugars comprise monosaccharides and disaccharides added to foods and beverages by manufacturers, cooks or consumers, together with sugars naturally present in honey, syrups, fruit juices and fruit juice concentrates [14]. In contrast, sugars naturally present in whole foods are typically less harmful to oral health, as they are consumed with fibres and nutrients that reduce their cariogenic potential [15]. In addition, Western diets rich in saturated fats and processed carbohydrates are associated with chronic inflammation and, consequently, an increased incidence of periodontal diseases [10]. Based on moderate-quality evidence, a strong recommendation supports limiting free sugar intake to less than 10% of total energy intake, while especially the focus on oral health underpins a conditional recommendation to further reduce intake to below 5% of energy intake [14]. Accordingly, lowering free sugar intake to these values can be recommended to improve health outcomes [5]. High intake of non-milk extrinsic sugars increases the risk of dental caries and is also linked to obesity and diabetes, which is why their reduction is recommended for both oral and general health [16]. Consistently, dental caries experience is lower in children and adults when free sugar intake is below the WHO recommendation, although the cariogenicity of other fermentable carbohydrates remains debated. Starches, particularly wheat-based grains, constitute a major component of Western diets and account for approximately 40–75% of total energy intake [7]. Despite the WHO recommendation, sugar consumption in industrialised regions like the European Union remains far above the recommended thresholds with approximately 60–120 g of total sugar intake [17].

There is evidence that the recommendation to avoid sugar has changed in dentistry in terms of its severity and strength, particularly due to the widespread introduction of fluoride in caries prevention. While the standard recommendation of dentists before World War II was a low-carbohydrate diet and vitamin D check, in the following decades, the central recommendation of fluoridated oral hygiene became increasingly dominant [18]. This shift in the prioritization of preventive approaches is particularly relevant in light of the common risk factor approach [16]. The concept of the common risk factor approach proposes that several chronic diseases share a limited number of modifiable behavioural risk factors. Excessive free sugar intake represents one of the most important examples of such shared risk factors because it contributes to both oral diseases and multiple non-communicable diseases. Consequently, dietary counselling delivered in dental practice may provide benefits extending far beyond oral health.

Umbrella reviews, which systematically evaluate and synthesize evidence from existing systematic reviews, provide an opportunity to concentrate an evidence-based summary of the overall strength, consistency, and quality of associations between sugar intake and health outcomes. To the best of the authors’ knowledge, there is currently only one existing umbrella review by Huang et al. that has summarised and analysed the negative health effects of regular sugar consumption [19]. The authors were able to include 73 studies, showing significant associations of sugar consumption with T2DM, body weight, obesity, metabolic syndrome, coronary heart disease (CHD), LDL-cholesterol, hypertension, and pancreatic cancer. However, their review mainly focused on metabolic outcomes and only included systematic reviews eligible for secondary quantitative analyses. Consequently, oral diseases, oral microbiome alterations and several additional systemic outcomes were only incompletely represented.

Therefore, the aims of this umbrella review were [1] to systematically summarise evidence from systematic reviews investigating sugar consumption and health [2], to compare evidence across oral and systemic health domains [3], to discuss the implications for preventive dentistry within the framework of the common risk factor approach supporting sugar reduction as a relevant public health and preventive strategy [9].

We hypothesised that higher dietary sugar intake, particularly free sugars and sugar-sweetened beverages, is consistently associated with adverse oral and systemic health outcomes across the available systematic review evidence.

## Materials and methods

### Study design

The umbrella review systematically searched relevant scientific databases for eligible studies, including Epistemonikos, PubMed, and the Cochrane Library, in accordance with Cochrane methodological criteria. There were no restrictions on study design, and included articles that investigated the intake of different types of sugars and reported on human health outcomes. Duplicates were removed both manually and using Covidence (Veritas Health Innovation Ltd, Australia). The systematic review was registered in the international register for systematic reviews Prospero (CRD42023380458).

For the literature analysis, a search strategy was developed to achieve the broadest possible inclusion of studies addressing the effects of sugar consumption. The search term “sugar” was applied across the MeSH term (keyword search), text word, and title/abstract fields. Combined using the Boolean operator “OR”, the resulting search string was:

- ((sugar[Text Word]) OR (sugar[Title/Abstract])) OR (sugar[MeSH Terms]). No filters or limits were applied.

### Eligibility criteria

To be included in the umbrella-review, studies were supposed to meet the following inclusion criteria:

- systematic review, with or without meta-analysis.
- focus on human populations.
- examination of any health outcome related to sugar intake.

Sugar could be investigated in various chemical forms, including monosaccharides, disaccharides, oligosaccharides, and polysaccharides; from different sources, including free and added sugars; and as part of dietary patterns involving sugar-sweetened beverages (SSB), soft drinks, sweets, chocolate, fruits, vegetables, or milk. Studies asserting the effects of honey and orange juice were included as well.

Exclusion criteria were:

- Primary studies (including randomized controlled trials, quasi-experimental studies, cohort studies, case–control studies, and cross-sectional studies).
- Studies lacking a clearly defined systematic methodology (reproducible literature search, predefined eligibility criteria and a structured evidence synthesis).
- Studies based on animal or in vitro models.
- Studies with insufficiently defined populations, interventions/exposures, comparators, outcomes, or study designs.
- Studies not reporting outcomes related to the effects of sugar consumption on human health.
- Studies where the full text was not accessible.
- Publications not available in English or German.
- Studies related to the pain relief in neonates (were excluded during outcomes assessment).

### Screening process, data extraction and evidence synthesis

The systematic literature search was conducted independently by two reviewers (ADZ, NW). During the first screening phase, studies were independently assessed based on their titles and abstracts and categorized as “include,” “exclude,” or “maybe.“. Regular meetings involving the entire study group (ADZ, NW, CT, and JPW) were held to discuss the review process and to resolve disagreements regarding study selection and methodological assessment. The screening process was supported by Covidence (Veritas Health Innovation Ltd., Australia), which was used to automatically remove duplicate records and to document the study selection process. In the second screening phase, full-text articles were assessed independently by both reviewers, and a final decision regarding study inclusion or exclusion was made. Within this process, grey literature and reference lists of the included reviews were also screened. After full texts were uploaded and assessed for eligibility, data extraction was performed. During data extraction, two reviewers (ADZ, NW) independently analysed primarily found studies. Disagreements were solved within the whole study group (ADZ, NW, CT, JPW).

Due to the broad scope of this umbrella review, no additional quantitative evidence synthesis was performed. Findings were synthesised narratively and all included studies were classified into predefined subtopics (based on [19]) according to health outcomes and reported effects, and these categories were expanded as necessary. Studies addressing more than one health outcomes were assigned to multiple sections. The various types of sugar listed in the inclusion criteria were noted in the results table and interpreted separately whenever possible. The study followed the Preferred Reporting Items for Systematic reviews and Meta-Analyses (PRISMA) 2020 protocol.

### Risk of bias analysis

The risk of bias analysis was conducted independently by two reviewers (ADZ and NW) using the AMSTAR-2 [20] (A Measurement Tool to Assess Systematic Reviews). Any discrepancies between reviewers were discussed and resolved by consensus. Afterwards, all the studies were categorized into four levels of confidence in quality according to AMSTAR-2 [20] classification.

## Results

**Fig. 1:**
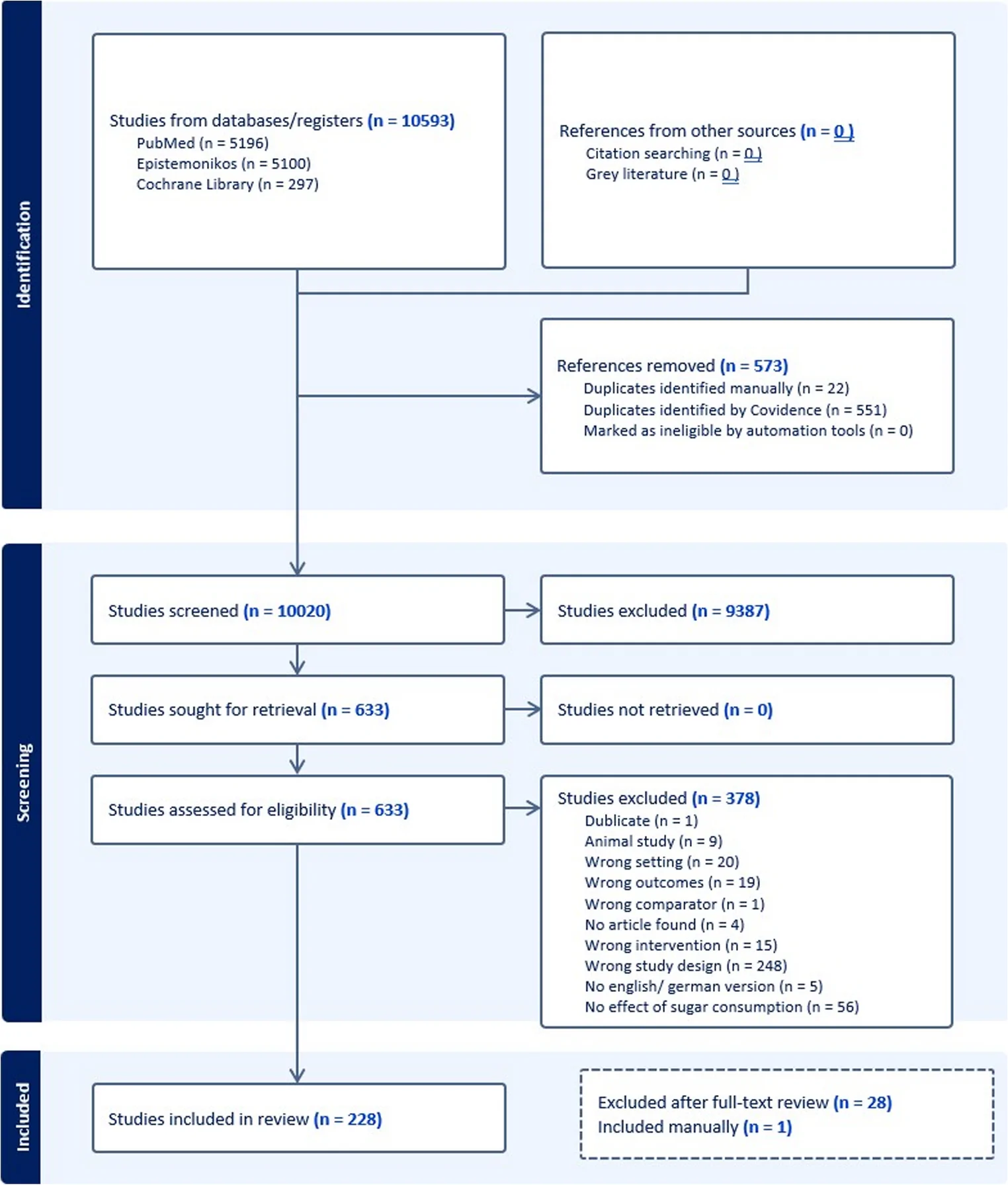
PRISMA flow diagram of the study selection process, including identification, screening, eligibility assessment, and final inclusion of systematic reviews

Data search conducted between July 2025 up to March 2026 revealed a total of 10,593 original articles, of which 9,387 were excluded during the title and abstract screening phase. Of the 633 studies assessed for full-text eligibility, 378 were excluded based on the predefined criteria, including insufficient study setting, outcomes, interventions or study design, use of animal models, lack of access to the full text, absence of English or German language versions, or insufficient reporting on the effects of sugar consumption on human health (see Fig. 1).

Inter-rater reliability between the two primary reviewers (ADZ and NW) showed a Cohen’s kappa (κ) of 0.492, indicating moderate agreement in screening decisions. In cases of uncertainty regarding study inclusion or exclusion, discrepancies were discussed among the reviewers, and consensus was reached through consultation with two other reviewers (CT and JPW).

**Fig. 2:**
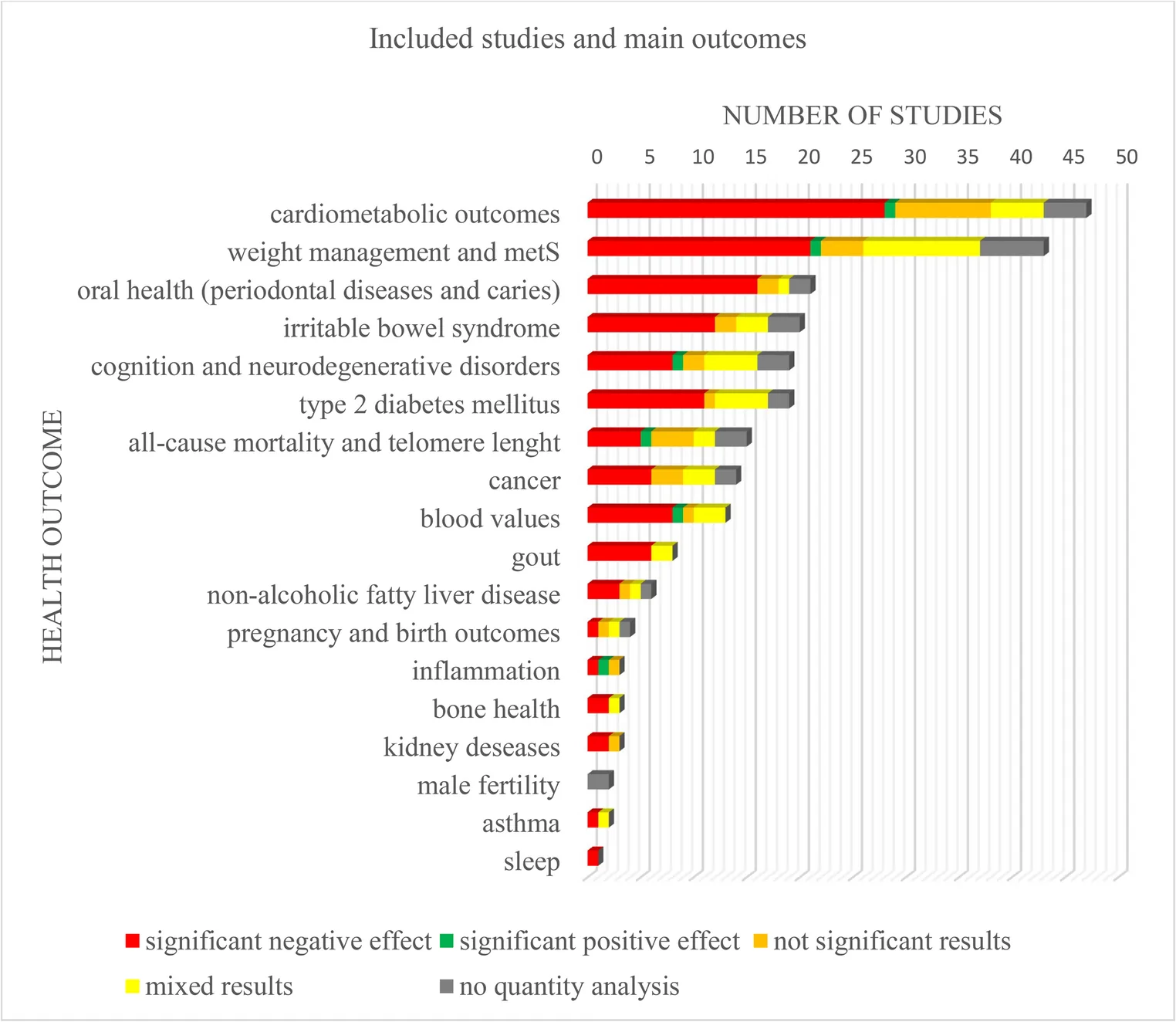
The total number of studies per health outcome and indicates how many reported significant positive, significant negative, non-significant, or mixed associations with sugar intake. Studies without quantitative analyses are also indicated. A few beneficial health outcomes were associated with fruit juice intake

Finally, a total of 228 included studies which met predefined eligibility criteria were included (Supplementary Table 1). The outcomes were categorized into 18 subtopics (metabolic disorders *n* = 95, reproduction *n* = 6, oral health *n* = 21, cancer *n* = 14, cardiometabolic risk factors *n* = 47, all-cause-mortality *n* = 15, kidney diseases *n* = 3, blood values *n* = 13, bone health *n* = 3, cognition and psyche *n* = 19, inflammation *n* = 3, asthma *n* = 2 and sleep *n* = 1) to provide the most comprehensive overview to the topic. The Fig. 2 illustrates the distribution of included studies across 18 subtopics, summarizing the types of effects of sugar intake reported in these studies. To the extent that detailed data were available, 132 (68%) studies examined sugar in the form of SSBs, 17 examined sugar in solid form, 12 examined sugar in form of juice, 13 examined fructose, and 20 examined FODMAPs.

### Metabolic disorders and weight management

Most of the studies (*n* = 95) focused on the association between sugar intake and metabolic or functional gastrointestinal disorders (type 2 diabetes mellitus (T2DM), gout, irritable bowel syndrome, non-alcoholic fatty liver (NAFLD) and weight management.

#### Type 2 diabetes mellitus

The research yielded 19 studies [21–39] which focused on the relationship between SSB intake and the risk of and T2DM. Ten studies demonstrated statistically significant positive association between high sugar-sweetened beverage (SSB) intake and T2DM, while two studies confirmed that excessive sugar consumption was associated with elevated blood glucose, insulin resistance, and higher incidence of diabetes [36, 37]. The incidence of T2DM per serving (250–355 ml) of SSB was associated with a 18% increase in normal weighted participants and by 13% in obese ones [31]. The dose-response analyses showed a 25% to 26% increased T2DM risk per serving (approx. 350 ml) per day [22, 29]. Several authors also concluded that metabolic syndrome and obesity were mediators between SSB intake and T2DM [22, 29, 33, 34]. Two articles identified a 21% increased risk of acquired insulin resistance per serving of SSB (250 ml), but no linear dose-response relationship was found [21, 33]. In summary, the included studies demonstrated a risk increase range of 13–31% per serving of SSB per day. Although no significant association was found for fruit juices. The available evidence was based on four studies [33–35, 38] with low and one study [36] with moderate AMSTAR-2 rating, 14 studies showed critically low quality (Supplementary Table 2). Table 1 shows the risk ratio of T2DM caused by SSB intake.

**Table 1:** Summary of systematic reviews reporting the association between sugar-sweetened beverage intake and the risk of type 2 diabetes mellitus (T2DM)

Study	Exposition	Risk ratio of T2DM
Toi et al. [21]	SSB	21–31%
Neuenschwander et al. [22]	SSB	26%
Schwingshackl et al. [33]	SSB	30%
Malik et al. [29]	SSB	26%
Meng et al. [32]	SSB	29%
Greenwood et al. [30]	SSB	20%
Imamura et al. [31]	SSB	13%−18%

#### Gout and hyperuricemia

A total of eight systematic reviews were found regarding the association between sugar consumption and gout or hyperuricemia [40–47]. All studies found that SSB and fructose consumption were linked to uric acid production. The highest fructose intake (> 11.8% to > 11.9% total energy) was associated with a 65% increased risk of gout development compared with the lowest intake (< 6.9% to < 7.5% total energy) [40]. The risk of gout increased by 28% [45] −77% [41] due to fruit juice intake and from 35% [46] to 108% [43] due to SSB intake, while there was no significant association between whole fruit intake and gout [43]. Evidence regarding uric acid levels was mixed, with some studies suggesting a possible increase, but no consistent harmful effect was demonstrated [47]. The highest SSB intake significantly increased the level of uric acid [41, 42, 45]. There was an overall significant positive association between SSB intake and increased risk of both hyperuricemia and gout. The available evidence was based on three studies [41, 46, 47] with low, one study [45] with moderate, and four studies [40, 42–44] with high AMSTAR-2 quality rating, no study showed critically low quality (Supplementary Table 2).

#### Irritable bowel syndrome

20 studies were found investigating sugar intake and irritable bowel syndrome (IBS) [48–67]. Eight studies confirmed that low-FODMAP (Fermentable Oligo-, Di-, Monosaccharides and Polyols) diets significantly reduced global IBS symptoms [48–50, 60, 61] and improved abdominal pain (OR = 1.81; [53, 58], bloating, and stool consistency [59]. Three reviews did not find any positive effect of low-FODMAP diets compared to habitual (including regular rye bread and no dietary education) diets [49, 51, 66]. Several reviews reported reduced levels of *Bifidobacterium* associated with a high-FODMAP diet, with no significant effects on gut microbial diversity or fecal short-chain fatty acids [55, 57]. High sugar intake was significantly associated with increased risk of ulcerative colitis (UC) by 59% [52, 65] and Crohn’s disease by 66% [65]. SSB intake was associated with Crohn’s disease by 58% and UC by 72% [65]. The available evidence was based on five studies [51–53, 58, 63] with low, two studies [50, 65] with moderate, and seven studies [48, 49, 56, 57, 59–61] with high AMSTAR-2 quality rating, five studies [54, 62, 64, 66, 67] showed critically low quality (Supplementary Table 2).

#### Non-alcoholic fatty liver disease

Six studies investigating the effects of sugar consumption on non-alcoholic fatty liver disease (NAFLD) were included [68–73]. A statistically significant positive association was found in all studies except one [69]. Three studies investigated the association between SSB intake and NAFLD and reported that SSB consumption increased the risk of NAFLD by 39% [68] and 40% [73] compared to non-SSB-consumers. Two meta-analyses found that intrahepatocellular lipids (IHCL) levels increased up to 54% on a hypercaloric high-fructose diet compared to a weight-maintaining diet [70, 71]. No change in liver values was recorded in isocaloric trials [69]. The available evidence was based on one study [71] with low AMSTAR-2 quality rating and five studies [68–70, 72, 73] showing critically low quality (Supplementary Table 2).

#### Weight management and metabolic syndrome

A total of 43 systematic reviews were found regarding the relationship between sugar consumption and different aspects of weight gain and weight management [34, 74–115]. Of these, 35 reviews analysed the association between SSB intake and obesity/overweight/body weight change across age groups and consistently confirmed a significant positive association. At least one additional serving (250 ml) of SSB per day significantly increased obesity risk up to 18% [75] and waist circumference (WC) by/up to 20% [75]. However, consuming two or more soft drinks per day was associated with an increased risk of obesity up to 50% [84]. The risk of increased body mass index (BMI) caused by SSB intake varied between 31% and 38% [76, 83]. Experimental studies found a weight gain of 1.6 kg over 10 weeks with daily sucrose consumption [81]. A significant positive association was found between SSB intake and higher body fat, whereas 100% fruit juice generally did not show this effect [89, 114]. Each SSB serving was associated to a 5% increase of obesity risk and a 12% risk of abdominal obesity [96]. When SSB intake continued for more than two weeks, it was linked to 0.04 kg/m^2^ BMI increase in 13-year-old children which contributed to increased body fat in early adulthood [94, 97]. Hypercaloric fructose intake was significantly associated with increased ectopic fat, particularly in the liver and muscles [100] and triglycerides [107]. Breastfeeding was concluded as a factor which was associated with a decreased obesity risk in adulthood [94], whereas early SSB exposure was linked to obesity in adult age [113]. High sugar snack foods were associated with overweight when combined with low vegetable intake [109]. In contrary, one study found that ready-to-eat cereals with added sugar were associated with lower BMI in some groups [112]. Six systematic reviews investigated the link between sugar consumption and metabolic syndrome. Metabolic syndrome (MetS) is defined as a cluster of major health risk factors such as cardiovascular disease and type 2 diabetes [34, 105]. The findings demonstrated that all SSB, total sweetened beverage (TSB), and ASB intake increased the risk of MetS by 56%, 51% and 44%, respectively [104]. Each additional 250 ml of SSB per day increased the risk of MetS by 19%−46% [104, 105]. Additional 335 ml of SSB per day increased the risk of MetS by 14%, whereas 100% fruit juice demonstrated a low risk of metabolic syndrome outcomes [108]. Table 2 shows the summary of systematic reviews reporting the association between SSBs, TSB intake and the risk of weight or metabolic related outcomes.

**Table 2:** Summary of systematic reviews reporting the association between sugar-sweetened beverages (SSBs), total sweetened beverages (TSBs) intake and the risk of weight or metabolic related outcomes

Study	Exposition	Type of outcome	Risk ratio
Qin et al. [74]	SSB	obesity	12%
Ruanpeng et al. [75]	SSB	obesity	18%
Ruanpeng et al. [75]	SSB	increased WC	20%
Malik et al. [81]	SSB	obesity	60%
Neelakantan et al. [83]	SSB	higher BMI	38%
Santos et al. [84]	SSB	obesity	17%−50%
Abbasalizad Farhangi et al. [85]	SSB	obesity	14%
Eugenia Pérez-Morales [90]	SSB	obesity in early childhood	4%
Schlesinger et al. [96]	SSB	overweight/obesity	20%
Schlesinger et al. [96]	SSB	abdominal obesity	12%
Schlesinger et al. [96]	SSB	gaining weight	23%
Te Morenga et al. [98]	SSB	overweight/obesity	55%
Yang et al. [99]	SSB	overweight/obesity	60%
Poorolajal et al. [101]	SSB	obesity	24%
Zhang et al. [104]	SSB	MetS	56%
Zhang et al. [104]	TSB	MetS	51%
Narain et al. [105]	SSB	MetS	46%
Semnani-Azad et al. [108]	SSB	MetS	14%

In conclusion, sugar intake, particularly from sugar-sweetened beverages, was consistently associated with weight gain, obesity, and an increased risk of metabolic syndrome. The available evidence was based on 14 studies [34, 74, 77, 82, 84, 85, 94, 95, 103, 104, 109, 111, 112, 114] with low, seven studies [80, 83, 91, 98, 101, 106, 110] with moderate, and four studies [93, 96, 107, 109] with high AMSTAR-2 quality rating, 18 studies [75–79, 81, 87–90, 92, 97, 99, 100, 105, 108, 113, 115] showed critically low quality (Supplementary Table 2).

### Reproduction and sugar intake

The umbrella review yielded six articles regarding the general topic reproduction and sugar intake. All articles can be organised in three subtopics: male fertility [116, 117], pregnancy and birth outcomes [118–121].

#### Male fertility

The umbrella review yielded two systematic reviews which investigated the association between sugar intake and fertility in men [116, 117]. Both highlighted the negative impact of excessive sugar consumption, particularly from sweets and SSBs in form of adversely affected sperm parameters and overall reproductive health [116]. Poor sperm quality (volume, vitality, motility, morphology, and concentration), hormonal levels, the number of spermatozoa and concentration were positively associated with a diet that included processed meat, soy foods, potatoes, full-fat dairy, total dairy products, cheese, coffee, alcohol, SSBs, and sweets. Higher SSB intake was associated with lower sperm motility, though it was unrelated to other semen quality parameters or reproductive hormone levels [117]. Regarding sugar, the study outcomes were limited because the study did not analyse the sugar effects separately. The available evidence was two studies [116, 117] with high AMSTAR-2 quality rating, no studies showed critically low quality (Supplementary Table 2).

#### Pregnancy and birth outcomes

The literature analysis identified four systematic reviews which investigated the effects of sugar consumption during pregnancy on birth weight and allergies [121]. Three of them included studies yielded inconsistent results. One study found higher birth weight [120], one found lower birth weight [119], and one found no correlation [118]. There was no significant association between high-sugary food or drink intake during pregnancy and obesity risk in offspring up to 18 years of age [120]. One study focused on prenatal sugar intake and childhood allergies and reported a weak, borderline 7% increase in asthma risk associated with maternal sugar intake during pregnancy [121]. Moreover, a potential risk of allergic rhinitis, eczema, wheezing and food intolerances was found [121]. One study investigated dietary patterns during pregnancy and potential birth outcomes, such as preterm birth, small-for-gestational-age, and birth weight [119]. Unhealthy dietary patterns, which included higher sugar consumption, were significantly associated with lower birth weight (mean difference: −40 g) and a 17% higher risk of preterm birth [119]. The available evidence was based on three studies [118, 120, 121] with moderate and one study [119] with high AMSTAR-2 quality rating, no studies showed low nor critically low quality (Supplementary Table 2).

### Cancer

The literature analysis revealed a total of 14 studies on the effects of sugar consumption on various cancers, as well as a study on the effects of high-sugar foods on cancer therapy [122–135].

Overall SSB intake was statistically significantly associated with increased general cancer risk by 12%, moreover each SSB serving per day increased the risk by 4% [129]. Fruit juice was linked to an increased overall risk of cancer by 14% [109]. Women who consumed no sugar-sweetened beverages compared with those who consumed more than 250 g SSB per day had a 27% lower risk of breast cancer [128]. The risk of prostate cancer increased by 14–18% with SSB intake [129, 130]. Due to insufficient data, none of the studies was able to demonstrate a significant association between endometrial/ovarian cancers and sugar consumption. Table 3 shows the association between sugar intake particularly with SSB and the risk of development of certain cancer types.

**Table 3:** Summary of systematic reviews reporting the association between sugar intake and the risk of different types of cancer

Study	Exposition	Type of cancer	Risk ratio
Li et al. [129]	SSB	total cancer risk	12%
Makarem et al. [128]	50-g increase in total sugar intake	total cancer risk	36%
Li et al. (2022)	SSB	colorectal carcinomas	14%
Makarem et al. [128]	total sugar intake	hepatocellular carcinomas	88%
Li et al. [129]	SSB	hepatocellular carcinomas	100%
Llaha et al. [130]	SSB	hepatocellular carcinomas	89%
Li et al. [129]	SSB	breast carcinomas	21%
Llaha et al. [130]	SSB	breast carcinomas	14%
Li et al. [133]	SSB	breast carcinomas	4%
Li et al. [129]	SSB	prostate carcinomas	14%
Llaha et al. [130]	SSB	prostate carcinomas	18%
Llaha et al. [130]	fruit juice	prostate carcinomas	3%
Carroll et al. [125]	fructose	colorectal carcinomas	30%

In summary, statistically significant positive associations between sugar consumption and an increased risk of certain cancers including pancreatic, colorectal, liver, breast and prostate cancer were observed. In particular, the consumption of SSBs and fruit juices appeared to increase the risk of cancer. However, the significance for individual cancer type was limited due to insufficient evidence. The available evidence was based on three studies [128, 130, 131] with moderate AMSTAR-2 quality rating, ten studies [122–127, 129, 130, 133] showed critically low quality. No studies were available in moderate or high quality (Supplementary Table 2).

### Cardiometabolic risk factors

The literature review identified a total of 47 studies on cardiometabolic and cardiovascular risk factors [32, 74, 83, 84, 136–178]. The risk ratio for cardiometabolic and cardiovascular outcomes associated with SSB consumption are shown in Table 4. Of these, 13 studies investigated the effects of sugar consumption on blood pressure. Eleven studies found a positive association between added sugar and hypertension [141, 146, 153, 157–162, 173, 178], and two did not [155, 170]. Consumption of SSB was associated with increased risk of hypertension by 6–21% [141, 146, 157–160, 175, 176]. Five studies found a statistically significant association between SSB intake and stroke risk, with an increase between 6% and 13% per serving [84, 154, 159, 166, 171]. Eleven studies associated SSB consumption with cardiovascular disease, of which ten studies found a statistically significant positive association [32, 74, 83, 145, 147, 149, 165, 167, 172, 179]. Cardiovascular mortality was found associated to increase between 8 and 24% due to high consumption of SSB [142]. Another study found a 17% higher risk of coronary heart disease, 7% for stroke, and 8% for heart failure per 250 ml SSB per day [172]. Findings regarding fruit juice consumption showed neutral, beneficial effects or no statistically significant associations [143, 148, 164, 169]. Overall, a statistically significant associations between fruit juice consumption and cardiovascular disease were observed. However, higher orange juice intake (> 500 ml/day) was associated to lower cholesterol levels and inflammation markers, whereas a statistically significant reduction in fasting blood glucose was found with cherry juice [143]. No effect was found for other factors such as LDL or HDL. Eight studies addressed the relationship between added/free sugars and the risk of cardiometabolic disease [136, 137, 139, 140, 144, 152, 163, 168], two of which found a positive association [137, 152]. In summary, 25 included studies show a statistically significant positive association between SSBs and the risk of hypertension, stroke, coronary heart syndrome, heart failure and cardiovascular mortality. The available evidence was based on 15 studies [74, 84, 141, 143, 150, 152, 155–157, 160, 162, 165, 169, 174, 175] with low, four studies [83, 136, 137, 168] with moderate, and three studies [138, 176, 178] with high AMSTAR-2 quality rating, 23 studies [32, 139, 142–149, 151, 153, 154, 158, 159, 161, 163, 164, 166, 167, 170, 171, 177] showed critically low quality (Supplementary Table 2).

**Table 4:** Summary of systematic reviews reporting the association between sugar intake and the risk of cardiometabolic outcomes

Study	Exposition	Cardiometabolic and cardiovascular outcome	Risk ratio
Cheungpasitporn et al. [161]	SSB	hypertension	12%
Kim and Je [157]	SSB	hypertension	8%−12%
Schwingshackl et al. [141]	SSB	hypertension	7%
Xi et al. [159]	SSB	hypertension	8%
Jayalath et al. [158]	SSB	hypertension	12%
Kim et al. [175]	SSB	hypertension	21%
Jamali et al. [176]	SSB	hypertension	13%
Malik et al. [160]	SSB	hypertension	6%
Santos et al. [84]	SSB	stroke	10%
Narain et al. [171]	SSB	stroke	13%
Wang et al. [166]	SSB	stroke	9%−12%
Xi et al. [159]	SSB	stroke	6%
Qin et al. [74]	SSB	hypertension	10%
Meng et al. [32]	SSB	cardiovascular disease	9%
Bechthold et al. [172]	SSB	coronary heart syndrome	17%
Kazemi et al. [165]	SSB	cardiovascular mortality	10%−16%
Sun et al. [179]	SSB	cardiovascular risk	8%−13%
Bhagavathula et al. [142]	SSB	cardiovascular mortality	6%−24%
Huang et al. [145]	SSB	coronary heart disease	17%
Khan et al. [144]	total sugars	cardiovascular mortality	9%
Khan et al. [144]	fructose	cardiovascular mortality	8%

### Cognition and psyche

A total of 19 systematic reviews were included evaluating the effect of sugar consumption and different cognitive performances or disorders, e.g. attention deficit hyperactivity disorder (ADHD), depression, anxiety Parkinson’s disease (PD), mood, and hippocampal function [166, 173, 180–196]. SSB consumption was significantly associated with a higher risk (17%) of cognitive disorders [189]. Two studies investigated the effects of sugar consumption on ADHD [181, 191]. Sugar and SSB consumption was significantly associated with an increased risk of ADHD symptoms by 22% [191]. Unhealthy dietary patterns or Western diets including refined cereals, high-fat dairy, desserts, sugar, sweets, and soft drinks were associated with a 41% increased risk of ADHD [181]. Sugar consumption was associated with an immediate improvement in mood, while its long-term effects were significantly detrimental to overall cognitive health [192]. The risk of all-cause dementia in middle-aged and older people increased 2.8-fold when consuming two and more servings of SSB per day [187]. Three studies provided dose-response meta-analyses which demonstrated similar results with an increased risk of depression by 5%−25% caused by SSB intake [173, 190, 195]. Two studies which investigated the association between SSB intake and anxiety did not find any statistically significant results [194, 196] and one confirmed higher SSB intake increased the risk of anxiety by 34% [193].

Summarized, higher sugar intake, especially from SSB, was associated with decreased cognitive performance, including executive function, attention, memory, and decision-making, and was associated with an increased risk of depression, attention deficit hyperactivity disorder and Parkinson’s disease. The available evidence was based on five studies [182, 189, 191, 192, 196] with low, two studies [183, 193] with moderate, and six studies [173, 181, 187, 188, 190, 197] with high AMSTAR-2 quality rating, six studies [166, 180, 184, 186, 194, 195] showed critically low quality (Supplementary Table 2).

### Other diseases and health outcomes

#### All-cause mortality and telomere length

Of the 15 included studies, eight systematic reviews examined the effects of consuming sugar-sweetened beverages (SSBs) or fruit juices [74, 165, 176, 198–202] and four examined the consumption of highly processed foods, sweets and added sugars in relation to all-cause mortality [163, 203–205]. All studies showed an increased risk in mortality associated with increased consumption of sugar. High consumption of SSB increased the all-cause mortality risk by 4–12% [74, 165, 176, 198, 199] and cardiovascular risk by 8–20% [199, 200, 204]. A daily sugar reduction of 5–40% or 100% substitution with sweeteners was associated with a 0.2–4% reduction in the prevalence of obesity and a 3.7–5.5% reduction in all-cause mortality from chronic diseases [206]. However, unlike SSB, no significant association was found for an equivalent amount of fruit juice consumption [201]. Two systematic reviews highlighted key dietary influences on telomere length in children and adolescents [207, 208]. There was a positive association between longer telomeres and the Mediterranean diet, higher intake of whole grains, coffee, and tea, along with a reduced intake of red meat. In contrast, the consumption of dairy products, sugars, SSBs, and refined cereals – particularly white bread – was associated with a 37% increased risk of telomeres shortening. Additionally, a diet with a high glycaemic load was negatively associated with telomere length. With regard to the current research question on the influence of sugar on general health, the included systematic reviews had limited informative value due cofounding nutritional factors. The available evidence was based on six studies [199–203, 205] with low and three studies [176, 207, 208] with high AMSTAR-2 quality rating, five studies [74, 163, 165, 198, 204] showed critically low quality (Supplementary Table 2).

#### Blood values

The current review yielded 13 studies that investigated blood value changes associated with sugar intake [103, 164, 178, 209–218]. The studies demonstrated that consumption of SSB and sweets were associated with increased levels of uric acid, whereas 100% fruit juice was associated with decreased uric acid levels [209]. There was a significant rise in postprandial triglycerides in hypercaloric trials linked to high fructose consumption [210, 217]. One study focusing on intermediate disease markers found that SSBs were associated with worsening lipid markers and increased risk coronary heart disease [211]. Furthermore, high SSB consumption was significantly associated with an increase in low-density lipoprotein cholesterol, decrease in high-density lipoprotein cholesterol and decrease in total cholesterol [213, 215].

The included studies showed that high sugar intake, particularly in the form of fructose and SSBs, were significantly associated with negative effects on lipid profiles, including increased LDL-C, decreased HDL-C, and increased triglycerides. The available evidence was based on three studies [103, 209, 219] with low and nine studies 1 [178, 211, 212, 214–219] with high AMSTAR-2 quality rating, one study [164] showed critically low quality (Supplementary Table 2).

#### Bone health

A total of three studies were found regarding sugar intake and bone health [220, 221]. The studies demonstrated that high sugar consumption, particularly SSB, was significantly associated with reduced bone health. A carbonated beverage or cola intake was linked to increase the fracture risk by 39% [220]. Increased consumption of SSB was associated with a significantly higher risk of hip fractures in men, while a modified Mediterranean Diet, which is low in sugar, demonstrated a decreased risk of hip fractures [222]. Higher SSB intake was associated with an 5–20% reduction in bone mineral density in adults [221]. In summary, SSB consumption was associated with an increased fracture risk in children and adults, lower bone mineral density, and lack of essential elements caused by milk replacement through SSB. The available evidence was based on one study [221] with low and two studies [203, 220] with moderate AMSTAR-2 quality rating, no studies showed critically low or high quality (Supplementary Table 2).

#### Inflammation

A total of three studies regarding sugar consumption and inflammation were found [223–225]. Fructose-containing sugars foods (SSB; sweetened dairy; sweetened dairy alternative [soy], 100% fruit juice, fruit, dried fruit, mixed fruit forms, sweetened cereal grains and bars, sweets and desserts, added nutritive sweetener, mixed sources with SSBs, and mixed sources without SSBs) did not demonstrate any significant influence on the level of C-reactive protein (CRP). However, some sources of fructose, such as sweetened soy beverages and 100% fruit juice, showed anti-inflammatory effects, while other sources, e.g. SSBs were associated with increased CRP levels [224]. Orange juice intake was associated with a significant decrease in the inflammatory cytokine IL-6 levels. However, markers such as C-reactive protein and malondialdehyde (MDA) and general oxidative stress did not show any significant decline [223].

In summary, the included studies showed a positive association between sugar consumption and increased levels of pro-inflammatory markers such as interleukin-6 (IL-6), and CRP. The available evidence was based on three studies [223–225] with high AMSTAR-2 quality rating, no studies showed critically low, low, nor moderate quality (Supplementary Table 2).

#### Kidney disease

The literature analysis revealed three studies on the effects of sugar consumption on kidney disease and urine production [226–228]. A constant or reduced urine volume was observed when 1 L of orange juice (10.5 g sugar/100 ml), cola (10.6 g sugar/100 ml) or sucrose (5–20 g sugar/100 ml) were consumed. The intake of 100–1000 mg honey per kilogram body weight caused an increase in urine volume [226]. The risk of chronic kidney disease was associated with an increase by 30%−58% linked to high SSB intake [227, 228]. In summary, the two included studies on chronic kidney disease showed a positive association with sugar consumption. However, the significance is limited due to the limited data available. The available evidence was based on one study [228] with moderate AMSTAR-2 quality rating, two studies [226, 227] showed critically low quality. No studies were available with high AMSTAR quality (Supplementary Table 2).

#### Sleep duration

One study investigated the relationship between sugar consumption and sleep [229]. Short sleep duration was defined as less than 7–8 h per night in adults and less than 9–10 h per night in children. An inverse relationship was found between sleep duration and consumption of SSB. Children with insufficient sleep duration consumed on average 16% more sugar, 18% more SSB, and 92% more energy drinks. In adults, a 58% increase in sugar consumption (including sugary energy drinks) was found to be significantly associated with shorter sleep duration. Since only cross-sectional studies were included in this study, a significant association was observed, but no conclusion on the causality of sugar consumption and sleep. The available evidence was based on one study [229] with moderate AMSTAR-2 quality rating, no studies showed critically low, low, nor high quality (Supplementary Table 2).

#### Asthma

The umbrella review yielded two systematic reviews regarding the relationship between SSB consumption and asthma [230, 231]. In adults, a meta-analysis of five studies found a significant associated increase in the prevalence of asthma by 37% with higher SSB intake. For children, ten studies indicated a 14% higher risk of asthma with high SSB consumption. SSB consumption was also significantly associated with a 9% increased risk of wheezing in children [230]. SSB intake was associated with an increased risk of asthma by 28%, while 100% fruit juice intake did not show any statistically significant association [231]. Overall, the included studies found that SSB consumption was significantly associated with an increased risk of asthma and respiratory issues in both children and adults. The available evidence was based on one study [231] with moderate, and one study [230] with high AMSTAR-2 quality rating, no studies showed critically low quality (Supplementary Table 2).

### Oral health

#### Caries and erosion

The literature review revealed 15 studies investigating the effects of sugar consumption on the dental hard tissues, such as caries or erosion [1–7, 164, 173, 232–237]. Apart from one study, all reviews found a significant positive association between sugar intake and caries. A significant positive association with higher caries risk was found for both sugary drinks and foods [1–5, 173, 232]. One study found a positive association between the consumption of processed sugary and starchy foods and caries incidence [7]. A 18% [236] − 57% increased risk of caries and 43% increased risk of erosion were found, when comparing moderate to low sugar consumption [4]. Moderate sugar consumers (1–2 SSB servings per day) showed a 22% higher DMFT value compared to low sugar consumers (0–1 SSB serving per day). In the same study, a further risk increase of 53% for caries and 209% for erosions was observed when comparing high with moderate sugar consumers. A strong increase in the risk of caries was found up to a daily intake of 150 ml of SSBs comparing to no intake [4]. One study reported that RCTs showed reduced enamel hardness and increased erosion with 100% fruit juice consumption, whereas prospective cohort studies found no associations with erosive or carious lesions [6]. Two studies focused on sugar intake and early childhood caries (ECC) and confirmed a strong statistically significant association [235, 237]. Sugar intake increases the risk of ECC by 224% [237].

In summary, a significant positive association was found between sugar consumption and caries and/or erosions. The available evidence was based on six studies [4, 5, 232, 235, 237, 238] with low, one study [3] with moderate, and three studies [6, 7, 173] with high AMSTAR-2 quality rating, five studies [1, 164, 233, 234, 236] showed critically low quality (Supplementary Table 2).

#### Periodontal diseases

Four studies on sugar consumption and periodontal disease were included [8–10, 15]. Three of these found a significant positive association, one did not find a significant association [10], however, the consumption of SSBs was associated to increase gingival bleeding and thus an increase in the risk of periodontitis. Therefore, the authors described added sugars from SSB as a risk factor in the development of periodontitis [8]. The findings from one study demonstrated a significant association between high-frequency intake of sugary drinks or foods and an increased incidence of periodontitis. A meta-analysis of the two included studies indicated an increased risk of periodontal disease [9]. Sugar reduction was associated with a significant reduction in gingival inflammation [15]. One study did not find any significant association between sugar consumption and periodontal diseases [10]. The available evidence was based on two studies [10, 15] with low and one study [8] with moderate AMSTAR-2 quality rating, one study [9] showed critically low quality (Supplementary Table 2).

#### Oral microbiome

Two reviews on the effects of sugar consumption on the oral microbiome were included, which found a significant positive association [11, 12]. Both reviews found a significantly associated reduced diversity of the oral microbiome under high sugar consumption and a predominance of certain species such as *Streptococci*, *Scardovia*, *Veillonella*, *Rothia*, *Actinomyces* and *Lactobacilli*. The available evidence was based on one study [11] with moderate AMSTAR-2 quality rating, one study [12] showed critically low quality (Supplementary Table 2).

Overall, the evidence consistently indicated that higher sugar consumption was associated with an increased risk of oral diseases, including caries, dental erosion, periodontal disease, and unfavourable changes in the oral microbiome, whereas sugar reduction appeared to have protective effects on oral health.

### Risk of bias analysis

**Fig. 3:**
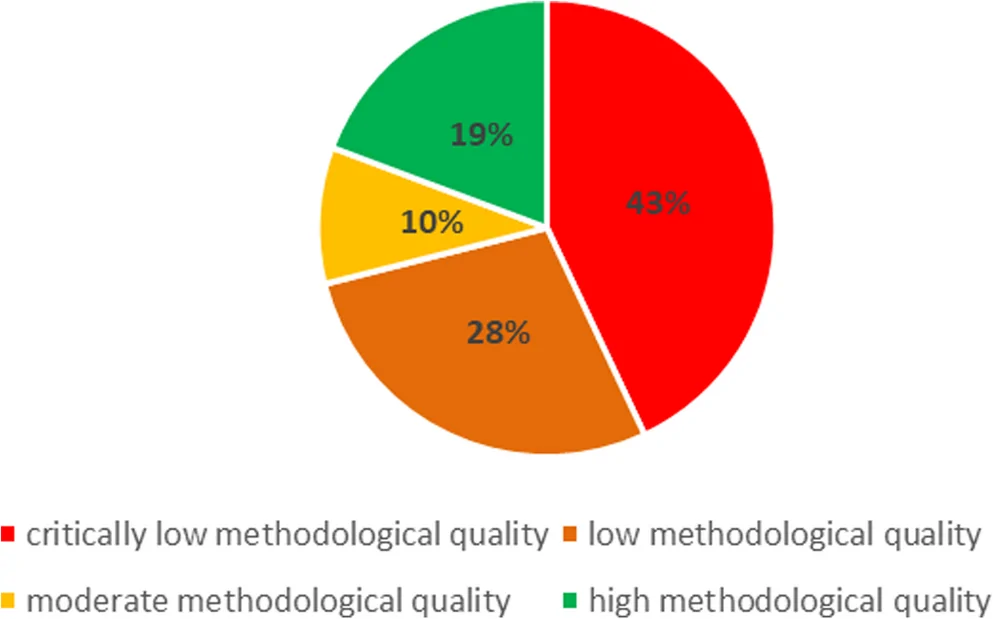
Risk of bias of the included studies assessed using AMSTAR-2

All included articles were evaluated using AMSTAR-2 [20]. The methodological quality assessment classified the included reviews into four categories: regarding methodological quality, 98 reviews were rated as critically low, 64 as low, 22 as moderate, and 44 as high according to AMSTAR-2 [20] which are represented in the Fig. 3. Overall, 71% of the reviews were classified as having low or critically low confidence in the results, indicating methodological weaknesses, whereas only 29% reached a moderate or high level of confidence.

## Discussion

**Fig. 4:**
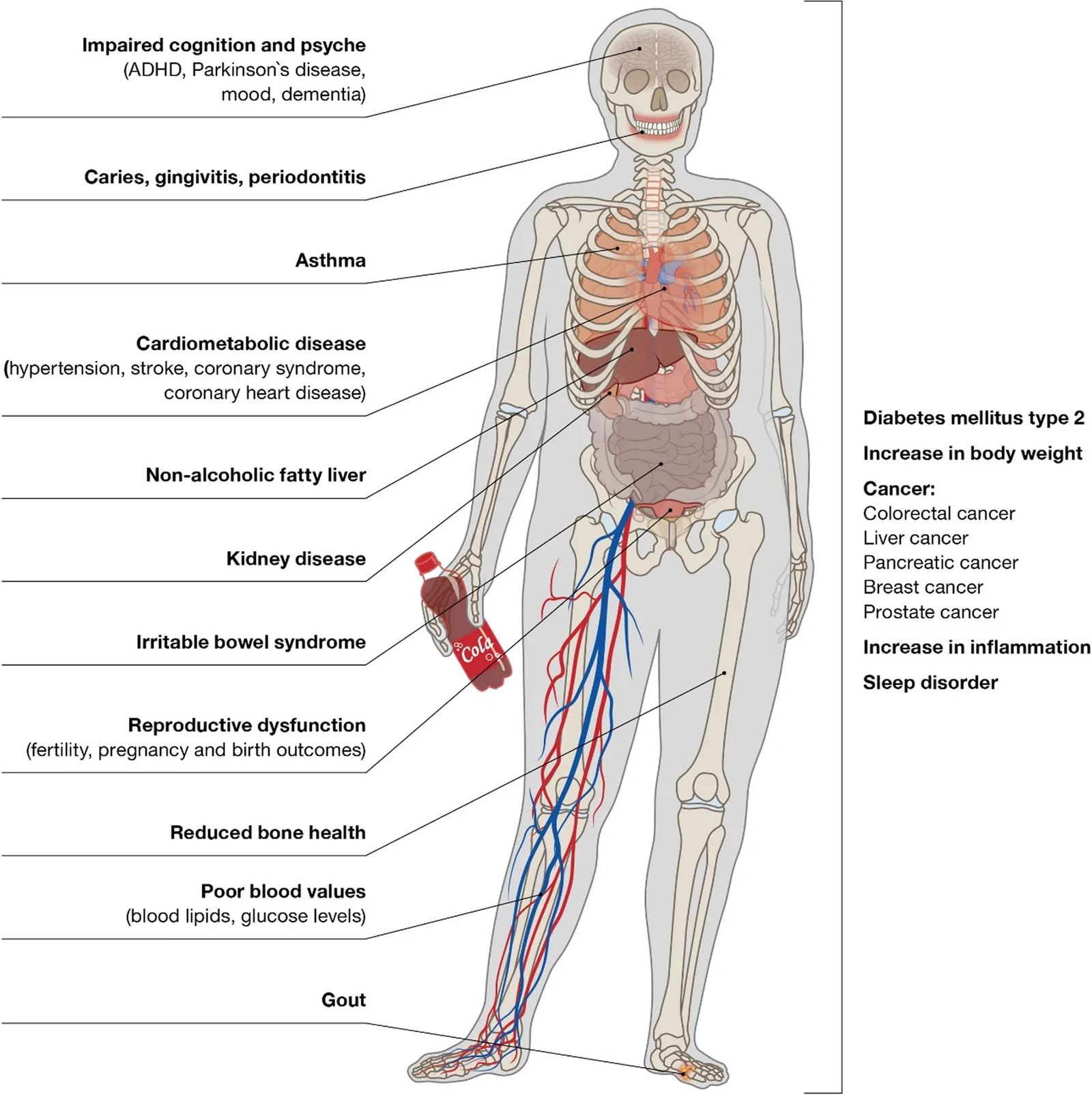
Illustration of the multisystem oral and general health outcomes associated with high sugar consumption found in this umbrella review

This umbrella review aimed to summarise the evidence from systematic reviews and meta-analyses regarding the effects of sugar consumption on oral and general health. The results revealed a consistent association between sugar consumption, particularly from SSBs, and a wide range of negative health outcomes, such as type 2 diabetes mellitus (T2DM), gout, non-alcoholic fatty liver disease (NAFLD), obesity, metabolic syndrome, dental caries, periodontal diseases, and increased all-cause mortality. Figure 4 summarizes these negative outcomes visually. The most consistent evidence with best AMSTAR-2 quality across the included reviews was found for cardiometabolic outcomes, particularly obesity-related measures and type 2 diabetes mellitus, which were repeatedly confirmed by several independent meta-analyses [19, 82, 150, 160]. Additionally, the associations with cardiovascular diseases, certain types of cancer and declined bone health, cognitive and mental health disorders were observed. However, the AMSTAR-2 quality rating revealed a need for further high-quality systematic reviews for outcomes regarding cancer, bone health, kidney diseases, sleep, non-alcoholic fatty liver disease, fertility, and the oral microbiome (Supplementary Table 2).

The findings of this umbrella review are consistent with the WHO [14] recommendations and the conclusions of Huang et al. [19], which indicate that high dietary sugar consumption is generally more harmful than beneficial for health and reduced intake is recommended to mitigate adverse outcomes. Reducing sugar consumption and limiting SSB intake further underline the need for both behavioural prevention (e.g. in kindergartens, schools, hospitals, nursing homes, dental practices, medical settings, workplaces) and structural prevention measures, such as sugar taxes and other policy regulations.

The included reviews repeatedly reported associations between sugar consumption and metabolic disorders as well as weight gain. High consumption of SSB was associated a significant increase in T2DM risk (13–31% per serving/day) and insulin resistance. Obesity or metabolic syndrome were described as mediators between the consumption of SSBs and T2DM [29, 34, 81, 205]. High blood glucose levels lead to increased levels of inflammation markers, such as C-reactive protein, which is also linked to the risk of diabetes [25]. Similarly, the positive association between SSB intake and NAFLD risk (~ 39–40%) can be explained by the fact that excessive fructose intake promotes hepatic triglyceride accumulation, leading to oxidative stress in hepatocytes and subsequent inflammatory responses in the liver [69]. Under conditions of excess energy intake, fructose-containing sugar-sweetened beverages may further promote de novo lipogenesis and intrahepatocellular fat accumulation [70, 71]. The primary mechanism by which increased sugar consumption promotes weight gain seems to be excessive calorie intake. Liquid sugars such as SSB may increase the risk of weight gain compared to solid foods because of reduced satiety of liquid sugary foods and intensified total energy intake [81]. The AMSTAR-2 rating showed several moderate to high quality studies in the field of metabolic disorders. However, especially in the field of T2DM and NAFLD additional high-quality studies would further strengthen the evidence base.

Metabolic syndrome is characterized by central obesity, hypertension, hyperglycaemia, elevated triglycerides, and low levels of HDL cholesterol [105]. Excess calorie intake, high fructose, ASB, or SSB consumption can lead to increased triglyceride synthesis, reduced insulin sensitivity, and hyperglycaemia. High fructose intake is associated with increased systolic and diastolic blood pressure and weight gain, particularly visceral obesity. All these factors are associated with unhealthy dietary patterns and lifestyles. Diet, especially high intake of fructose, seems to play a key role [104–106, 108].

The authors of several systematic reviews described different pathomechanisms of inflammation as a response to sugar consumption. Sugar, particularly fructose, lead to metabolic processes that initiate increased production of uric acid in the liver and free fatty acids, which can activate immunological responses as well as oxidative stress [223–225]. The increased level of uric acid activates reactive oxygen species, which, can cause damage to cellular structures. Regular sugar intake is associated with chronic inflammatory conditions such as type 2 diabetes, IBS and cardiovascular disease. Moreover, sugar intake is associated with gut microbiome disbalance, specifically, decreased microbiotic diversity and associated with an increased amount of harmful bacteria, which initiate the production of lipopolysaccharides, increased intestinal permeability, immune system activation, another trigger of high cytokine production [67].

High sugar intake seems to negatively affect sperm quality through increased blood sugar and insulin production. In long-term, this may lead to insulin resistance and affect the hypothalamic-pituitary-gonadal (HPG) axis, which regulates male reproductive function and leads to imbalance in the testosterone-estrogen ratio in the body. Furthermore, increased sugar consumption was found to be associated with higher production of reactive oxygen species, which may lead to DNA damage in sperm and to lower sperm production [116, 117]. Beyond this, high sugar intake may accelerate biological ageing by increasing oxidative stress and chronic inflammation, which are associated with accelerated telomere shortening and reduced cellular longevity [207, 208].

The significant association of sugar consumption to cancer forms such as pancreatic [128, 130, 198], colorectal [128, 198], liver [130, 198], breast [130, 198], and prostate cancers [130, 198] was a major finding of the umbrella review. The range of the increase in risk was strongly depending on the type of cancer and the sugar source (total cancer risk varied between 12 and 36%). A link between colorectal cancer and sugar or sugar-sweetened beverage consumption may be mediated by increased insulin production and the associated accumulation of visceral adipose tissue leading to obesity. Insulin is a growth factor for colon cells and increases the risk of colorectal cancer. Patients with gastric cancer showed increased IGF-1 levels compared to healthy patients [135]. However, there seem to be still a need of high-quality systematic reviews in this field to strengthen the evidence base.

Higher sugar intake, especially from SSB, was associated with poorer cognitive performance, increased risk of ADHD, depression, and neurodegenerative diseases. The included studies suggested that SSB and sugar consumption were associated with anxiety, depression, stress, and negative emotions. Moreover, there was a bidirectional relationship between depression and obesity and between depression and type 2 diabetes [173]. The possible pathomechanism of anxiety/depression and soft drink consumption can be explained by endothelial dysfunction or a higher level of inflammation. Furthermore, glucose boosts the production and release of acetylcholine in the hippocampus which may also lead to gamma-aminobutyric acid release. High sugar consumption seemed to provoke gastrointestinal discomfort or reactive hypoglycemia and activated neurotransmitter functions and seems to be linked to a lack of essential nutrients such as iron and zinc. All these factors are known to worsen ADHD symptoms [181, 191].

SSB consumption was associated with lower bone mineral density and increased fracture risk because of increased urinary calcium loss and bone mineralization disorders. The phosphoric acid in SSB can also impair calcium absorption, raising the risk of fractures and osteoporosis. Additionally, higher levels of C-reactive protein may lead to increased bone resorption and chronic inflammation [220–222]. With regard to the high prevalence of both osteoporosis and high sugar consumption, more high-quality systematic reviews are needed. SSB intake may also significantly increase the risk of asthma. Regarding the underlying mechanisms, sugar has been shown to trigger inflammatory reactions which are associated with C-reactive protein and additives such as sodium benzoate and sulfites, which could also play a part in asthma development.

Based on the presented associations between sugar intake and multiple chronic diseases, it is not surprising that sugar consumption was also linked to increased all-cause mortality (increased risk ~ 4–12%), particularly through its contribution to cardiovascular risk. High sugar intake negatively affected lipid profiles, increasing LDL, decreasing HDL, and raising triglycerides [103, 213]. High consumption of SSBs and ASBs may contribute to increased mortality risk, through cardiovascular mechanisms. This highlights the potential importance of limiting sweetened beverage intake as part of strategies to reduce long-term health risks [198]. Because of their high caloric content, moderate-to-high glycemic index, and low satiety effect, increased intake of sugar-sweetened beverages is associated with weight gain, insulin resistance, inflammation, and atherogenic dyslipidemia [200]. Additionally, both sugar- and artificially sweetened beverages may contribute to metabolic disturbances through appetite dysregulation, altered gut microbiota, potentially leading to excess energy intake and increased cardiometabolic and mortality risk (~ 8–24% increased risk) [74].

Finally, the results of this umbrella review demonstrated a consistent association between higher intake of free sugar and adverse oral health outcomes, including dental caries (~ 18–43% increased risk), ECC, dental erosion, and periodontal diseases. The risk increased proportionally with sugar consumption levels. The frequent intake of sugar, especially more than 5% of the daily energy intake, was associated with a higher risk of caries and dental erosion [5–7, 232]. Dental caries arises when oral bacteria metabolise sugars into acids that lower biofilm pH below the critical threshold for enamel demineralisation. Sugar-sweetened beverages further increase caries risk by combining high sugar content with intrinsic acidity, leading to sustained tooth surface loss [4]. Early childhood caries (ECC) is common worldwide in preschool children and can negatively affect health, nutrition, and quality of life [237]. Sugar intake can promote gingivitis by increasing plaque accumulation and by bacterial sugar metabolism into pro-inflammatory acids that irritate the gingiva. In addition, systemic effects of free sugars, such as hyperglycaemia and impaired immune responses, may further increase gingival inflammation [15]. Periodontal diseases were also linked to sugar intake, possibly due to its effect on chronic gingival inflammation, impaired bone metabolism, which together increase susceptibility to alveolar bone loss and microbiome changes [10]. Due to the production of adhesins and polysaccharides, the bacteria easily attach to the tooth surface, making them one of the triggers of dental caries. The authors described *Streptococci* as one of the dominant genera in the oral microbiome [11, 12]. While the evidence base and the quality of studies on dental caries with 15 included studies can be considered robust, more high-quality systematic reviews on periodontal diseases and the oral microbiome are needed, as only four and two studies could be included, respectively.

### Implications for oral and general health, and dental professionals

Oral diseases seem to represent the earliest and most consistently documented health consequences of excessive sugar consumption. In contrast to many presented chronic non-communicable diseases, which often develop over decades, adverse effects on oral tissues—including dental caries, alterations of the oral microbiome and periodontal inflammation—may occur within relatively short periods of sustained high sugar intake. Consequently, oral diseases may serve as early clinical indicators of an unhealthy dietary pattern. While the association between free sugar intake and dental caries has long been established, the present umbrella review demonstrates that the potential oral consequences of excessive sugar consumption extend beyond caries alone. The available evidence further indicates that dietary sugar may contribute to gingival and periodontal inflammation, although the certainty of evidence is lower than for dental caries. Experimental and clinical studies suggest that excessive free sugar intake may promote a pro-inflammatory environment through systemic metabolic effects, altered immune responses and microbiome-mediated mechanisms. However, the methodological quality of the currently available systematic reviews remains heterogeneous, and further high-quality prospective studies are required to clarify these associations.

**Fig. 5:**
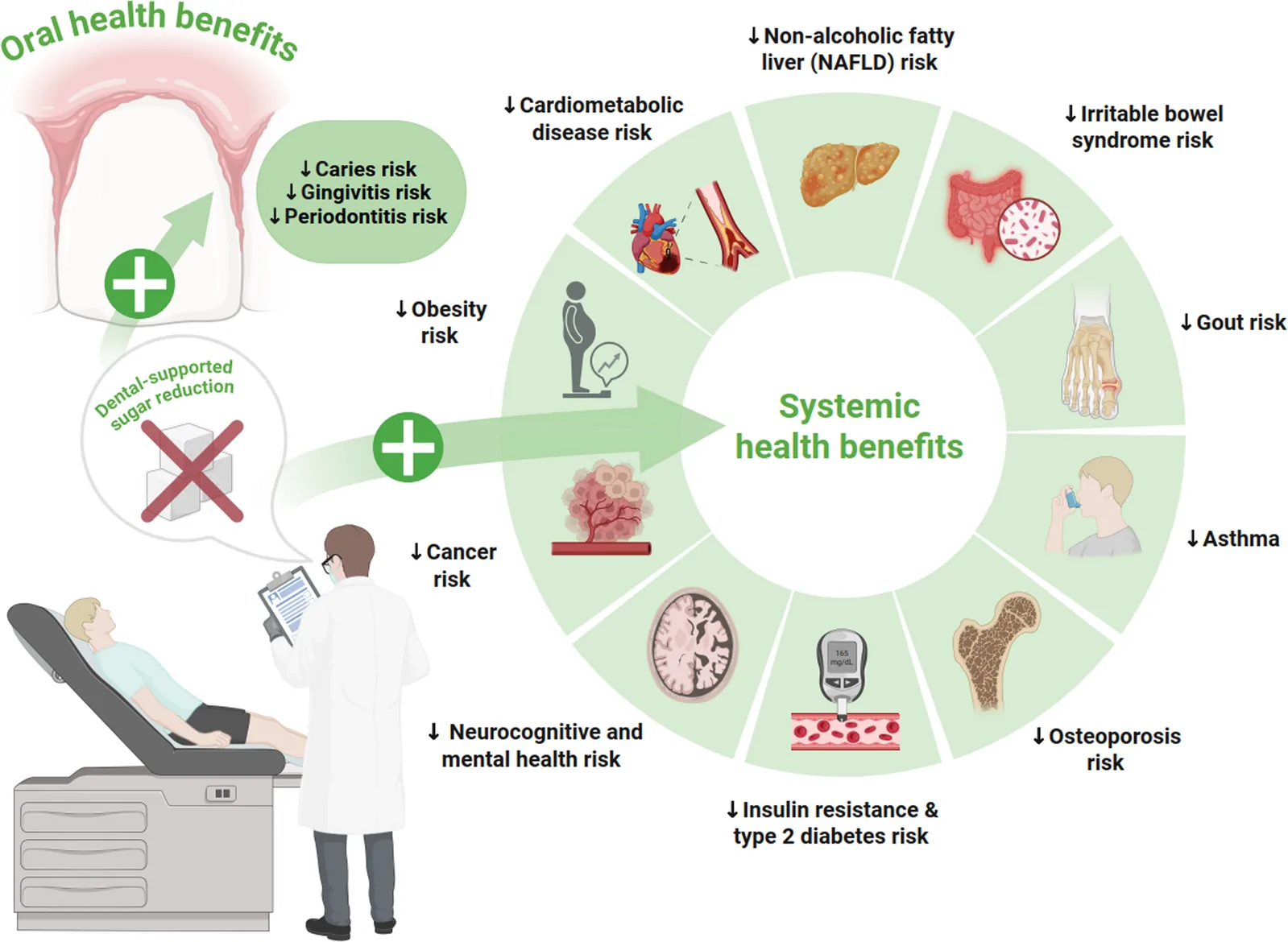
Shared health effects of a sugar reduction promoted by the dental professional based on the findings of the umbrella review

From a clinical perspective, these findings reinforce the central role of dental professionals in dietary counselling. Because dental practitioners routinely examine patients throughout life and frequently identify early manifestations of sugar-related diseases, they are uniquely positioned to promote dietary modification before systemic complications become clinically apparent. Integrating dietary counselling into routine dental care therefore represents an important opportunity to simultaneously improve oral health and contribute to the prevention of chronic non-communicable diseases within the framework of the common risk factor approach (Fig. 5).

### Strengths and Limitations

Most included studies confirmed the adverse health effects of sugar consumption in its multiple forms. Most of the investigations were related to SSB consumption. However, during data extraction, particularly in relation to sugar-specific exposures, it became apparent that several studies (excluding those investigating neonatal analgesia) examined sugar intake only as a part of dietary patterns. In consequence, the reported negative outcomes are likely related not only to sugar, but to a cluster of lifestyle factors, including consumption of SSB and ASB, excessive caloric-, processed foods- and saturated fats, lower level of physical activity, and increased screen time. These factors may represent a generalized lifestyle pattern, often related to the populations with lower health awareness. Additionally, under real-life conditions, accurately quantifying individual sugar intake remains methodologically challenging.

It should be noted that two reviews [69, 71] reported conflicts of interest with beverage/food manufacturers (e.g. Coca-Cola, Dr. Pepper Snapple Group, Unilever), the Canadian Sugar Institute or the International Life Sciences Institute (ILSI). Some studies were funded by The Peanut Institute, Barilla, Unilever, Unico, Primo, Loblaw Companies, Quaker (PepsiCo), Pristine Gourmet, Bunge Limited, Kellogg Canada, WhiteWave Foods [209], Unilever, a commercial food and beverage manufacturer [61]. Liska et al. [6] stated cooperation with juice product associations but found a positive association of fruit juice consumption with erosion in their review. Seven studies regarding cardiometabolic risk factors reported conflicts of interest with the sugar industry or associations such as the World Sugar Research Organization [139, 140, 144, 146, 155, 158, 165, 170]. It is striking that, except for the review by Gibson et al., all studies involved the same authors with a conflict of interest. Of these seven studies with conflict of interest, four found no positive associations, while Jayalath et al., Kazemi et al. and Liu et al. found positive associations of sugar-sweetened beverages with cardiovascular disease.

Several methodological limitations should be considered. First, several included systematic reviews were rated as low or critically low according to AMSTAR-2. Second, overlap of primary studies between reviews was not formally quantified and may therefore have increased the apparent amount of evidence. Third, exposure definitions and heterogeneity varied considerably across reviews, including free sugars, added sugars, sugar-sweetened beverages, total sugars and dietary patterns. Fourth, as this umbrella review aimed to summarise and compare findings from published systematic reviews rather than to re-evaluate the certainty of evidence for individual associations, no additional GRADE assessment was conducted. Instead, the methodological quality of the included systematic reviews was assessed using the AMSTAR-2 tool. For further meta-analysis we refer to Huang et al. [19]. Finally, because most underlying evidence originated from observational studies, causal conclusions cannot be drawn. The search results were limited to English and German publications.

## Conclusions

High sugar intake, particularly from sugar-sweetened beverages, was consistently associated with an increased risk of oral diseases such as dental caries, gingivitis and periodontitis, as well as systemic conditions including obesity, type 2 diabetes mellitus, cardiovascular diseases, non-alcoholic fatty liver disease, and metabolic syndrome. These findings indicate the clinical relevance of sugar reduction for maintaining oral health, underlining the specific preventive role of dentistry through early identification, patient education, and support for reducing free sugar intake. Consequently, limiting sugar consumption constitutes a key strategy not only for improving oral health but also for contributing to comprehensive public health prevention efforts. Dental professionals help prevent disease by supporting patients in reducing sugar intake, which is important for both oral and general health.

## Supplementary Information

Below is the link to the electronic supplementary material.

Supplementary Material 1

Supplementary Material 2

Supplementary Material 3

## Data Availability

The results of the literature search and risk of bias assessment are available in the supplementary material.

## References

[1] Anderson CA, Curzon MEJ, Van Loveren C, Tatsi C, Duggal MS (2009) Sucrose and dental caries: a review of the evidence. Obes Rev. 10.1111/j.1467-789X.2008.00564.x 19207535

[2] Moynihan P (2016) Sugars and dental caries: evidence for setting a recommended threshold for intake. Adv Nutr. 10.3945/an.115.009365 PMC4717883 26773022

[3] Baghlaf K, Muirhead V, Moynihan P, Weston-Price S, Pine C (2018) Free sugars consumption around bedtime and dental caries in children: a systematic review. JDR Clin Translational Res 3(2):2. 10.1177/2380084417749215 30931774

[4] Valenzuela MJ, Waterhouse B, Aggarwal VR, Bloor K, Doran T (2021) Effect of sugar-sweetened beverages on oral health: a systematic review and meta-analysis. Eur J Public Health. 10.1093/eurpub/ckaa147 32830237

[5] Mahboobi Z, Pakdaman A, Yazdani R, Azadbakht L, Montazeri A (2021) Dietary free sugar and dental caries in children: a systematic review on longitudinal studies. Health Promot Perspect 11(3):271–280. 10.34172/hpp.2021.35 PubMed PMID: 34660221; PubMed Central PMCID: PMC8501477 PMC8501477 34660221

[6] Liska D, Kelley M, Mah E (2019) 100% fruit juice and dental health: a systematic review of the literature. Front Public Health 7:190. 10.3389/fpubh.2019.00190 PMC6640211 31355175

[7] Hancock S, Zinn C, Schofield G (2020) The consumption of processed sugar- and starch-containing foods, and dental caries: a systematic review. Eur J Oral Sci 128(6):467–75. 10.1111/eos.12743 33156952

[8] Gupta V, Dawar A, Bhadauria US, Purohit BM, Nilima N (2023) Sugar-sweetened beverages and periodontal disease: a systematic review. Oral Dis 29(8):3078–3090. 10.1111/odi.14368 PubMed PMID: 36062371 36062371

[9] Kusama T, Nakazawa N, Takeuchi K, Kiuchi S, Osaka K (2022) Free sugar intake and periodontal diseases: a systematic review. Nutrients 14(21):4444 doi:10.3390/nu14214444 PubMed PMID: 36364708; PubMed Central PMCID: PMC9656760 10.3390/nu14214444 PMC9656760 36364708

[10] Jeong J, Kim HS, Lee D, Kim K, Kim YH (2022) Association between four dietary patterns and the risk of periodontal diseases: a systematic review and meta-analysis. Nutrients 14(20):4362. 10.3390/nu14204362 PMC9611912 36297045

[11] Angarita-Díaz MDP, Fong C, Bedoya‐Correa CM, Cabrera‐Arango CL (2022) Does high sugar intake really alter the oral microbiota?: a systematic review. Clin Exp Dent Res. 10.1002/cre2.640 PMC9760141 35946056

[12] Dhingra K, Jeng JH (2023) Does a high-sugar diet alter the bacterial diversity of the oral cavity? Evid Based Dent 24(1):1. 10.1038/s41432-023-00862-y 36890244

[13] Machiulskiene V, Campus G, Carvalho JC, Dige I, Ekstrand KR, Jablonski-Momeni A et al (2020) Terminology of dental caries and dental caries management: consensus report of a workshop organized by ORCA and Cariology Research Group of IADR. Caries Res 54(1):7–14. 10.1159/000503309 31590168

[14] World Health Organization (2015) Guideline: sugars intake for adults and children. World Health Organization 25905159

[15] Woelber JP, Gebhardt D, Hujoel PP (2023) Free sugars and gingival inflammation: a systematic review and meta-analysis. J Clin Periodontology 50(9):1188–1201. 10.1111/jcpe.13831 37246336

[16] Sheiham A, Watt RG (2000) The Common Risk Factor Approach: a rational basis for promoting oral health. Comm Dent Oral Epid 28(6):399–406. 10.1034/j.1600-0528.2000.028006399.x 11106011

[17] EFSA Panel on Nutrition, Novel Foods and Food Allergens (NDA), Turck D, Bohn T, Castenmiller J, de Henauw S, Hirsch-Ernst KI et al (2022) Tolerable upper intake level for dietary sugars. EFSA J 20(2):e07074. 10.2903/j.efsa.2022.7074 PMC8884083 35251356

[18] Hujoel PP (2022) Private interests and the start of fluoride-supplemented high-carbohydrate nutritional guidelines. Nutrients 14(20):4263. 10.3390/nu14204263 PMC9610923 36296949

[19] Huang Y, Chen Z, Chen B, Li J, Yuan X, Li J et al (2023) Dietary sugar consumption and health: umbrella review. BMJ 381:e071609. 10.1136/bmj-2022-071609 PMC10074550 37019448

[20] Shea BJ, Reeves BC, Wells G, Thuku M, Hamel C, Moran J et al (2017) AMSTAR 2: a critical appraisal tool for systematic reviews that include randomised or non-randomised studies of healthcare interventions, or both. BMJ. 10.1136/bmj.j4008 PMC5833365 28935701

[21] Toi PL, Anothaisintawee T, Chaikledkaew U, Briones JR, Reutrakul S, Thakkinstian A (2020) Preventive role of diet interventions and dietary factors in type 2 diabetes mellitus: an umbrella review. Nutrients 12(9):9. 10.3390/nu12092722 PubMed PMID: 32899917; PubMed Central PMCID: PMC7551929 PMC7551929 32899917

[22] Neuenschwander M, Ballon A, Weber KS, Norat T, Aune D, Schwingshackl L et al (2019) Role of diet in type 2 diabetes incidence: umbrella review of meta-analyses of prospective observational studies. BMJ 366:l2368. 10.1136/bmj.l2368 PMC6607211 31270064

[23] Sievenpiper JL, Carleton AJ, Chatha S, Jiang HY, de Souza RJ, Beyene J et al (2009) Heterogeneous effects of fructose on blood lipids in individuals with type 2 diabetes. Diabetes Care. 10.2337/dc09-0619 PMC2752906 19592634

[24] D’Arcy E, Rayner J, Hodge A, Ross LJ, Schoenaker DAJM (2020) The role of diet in the prevention of diabetes among women with prior gestational diabetes: a systematic review of intervention and observational studies. J Acad Nutr Diet. 10.1016/j.jand.2019.07.021 31636052

[25] Esposito K, Kastorini CM, Panagiotakos DB, Giugliano D (2010) Prevention of type 2 diabetes by dietary patterns: a systematic review of prospective studies and meta-analysis. Metab Syndr Relat Disord 8(6):6. 10.1089/met.2010.0009 20958207

[26] Bellou V, Belbasis L, Tzoulaki I, Evangelou E (2018) Risk factors for type 2 diabetes mellitus: an exposure-wide umbrella review of meta-analyses. PLoS One 13(3):3. 10.1371/journal.pone.0194127 PMC5860745 29558518

[27] Xi B, Li S, Liu Z, Tian H, Yin X, Huai P et al (2014) Intake of fruit juice and incidence of type 2 diabetes: a systematic review and meta-analysis. PLoS One. 10.1371/journal.pone.0093471 PMC3969361 24682091

[28] Tsilas CS, de Souza RJ, Mejia SB, Mirrahimi A, Cozma AI, Jayalath VH et al (2017) Relation of total sugars, fructose and sucrose with incident type 2 diabetes: a systematic review and meta-analysis of prospective cohort studies. CMAJ. 10.1503/cmaj.160706 PMC5436961 28536126

[29] Malik VS, Popkin BM, Bray GA, Després JP, Willett WC, Hu FB (2010) Sugar-sweetened beverages and risk of metabolic syndrome and type 2 diabetes. Diabetes Care. 10.2337/dc10-1079 PMC2963518 20693348

[30] Greenwood DC, Threapleton DE, Evans CEL, Cleghorn CL, Nykjaer C, Woodhead C et al (2014) Association between sugar-sweetened and artificially sweetened soft drinks and type 2 diabetes: systematic review and dose–response meta-analysis of prospective studies. Br J Nutr 112(5):5. 10.1017/S0007114514001329 24932880

[31] Imamura F, O’Connor L, Ye Z, Mursu J, Hayashino Y, Bhupathiraju SN et al (2015) Consumption of sugar sweetened beverages, artificially sweetened beverages, and fruit juice and incidence of type 2 diabetes: systematic review, meta-analysis, and estimation of population attributable fraction. BMJ. 10.1136/bmj.h3576 PMC4510779 26199070

[32] Meng Y, Li S, Khan J, Dai Z, Li C, Hu X et al (2021) Sugar- and artificially sweetened beverages consumption linked to type 2 diabetes, cardiovascular diseases, and all-cause mortality: a systematic review and dose-response meta-analysis of prospective cohort studies. Nutrients 13(8):8. 10.3390/nu13082636 PubMed PMID: 34444794; PubMed Central PMCID: PMC8402166 PMC8402166 34444794

[33] Schwingshackl L, Hoffmann G, Lampousi AM, Knüppel S, Iqbal K, Schwedhelm C et al (2017) Food groups and risk of type 2 diabetes mellitus: a systematic review and meta-analysis of prospective studies. Eur J Epidemiol. 10.1007/s10654-017-0246-y PMC5506108 28397016

[34] Banjarnahor RL, Javadi Arjmand E, Onni AT, Thomassen LM, Perillo M, Balakrishna R et al (2025) Umbrella review of systematic reviews and meta-analyses on consumption of different food groups and risk of type 2 diabetes mellitus and metabolic syndrome. J Nutr 155(5):1285–1297. 10.1016/j.tjnut.2025.03.021 PMC12121416 40122387

[35] Choi JH, Song S, Kim SK, Cho JW, Bae JH, Moon S et al (2026) Health effects of sugar-sweetened and artificially sweetened beverages: umbrella review and evidence-based consensus statement of the Korean diabetes association and the Korean nutrition society. Diabetes Metab J 50(1):32–46. 10.4093/dmj.2025.0848 PMC12813387 41531289

[36] Della Corte KA, Bosler T, McClure C, Buyken AE, LeCheminant JD, Schwingshackl L et al (2025) Dietary sugar intake and incident type 2 diabetes risk: a systematic review and dose-response meta-analysis of prospective cohort studies. Adv Nutr 16(5):100413. 10.1016/j.advnut.2025.100413 PMC12145082 40122386

[37] Díaz-Benavides DA, Muhlis ANA, Chamouni G, Charles R, Nigatu DT, Ben Khadra J et al (2025) Nutritional determinants of type 2 diabetes mellitus in the European Union: a systematic review. Nutrients 17(22):3507. 10.3390/nu17223507 PMC12655033 41305558

[38] Guntari P, Junaida A, Eny P, Pichamon N, Wiroj J, Suwimol S (2025) Fructose-containing sugars and metabolic risk: a systematic review and meta-analysis. Food Nutr Res. 10.29219/fnr.v69.11062 PMC12664302 41323133

[39] Jannasch F, Kröger J, Schulze MB (2017) Dietary patterns and type 2 diabetes: a systematic literature review and meta-analysis of prospective studies. J Nutr 147(6):1174–82. 10.3945/jn.116.242552 28424256

[40] Jamnik J, Rehman S, Blanco Mejia S, De Souza RJ, Khan TA, Leiter LA et al (2016) Fructose intake and risk of gout and hyperuricemia: a systematic review and meta-analysis of prospective cohort studies. BMJ Open 6(10):e013191. 10.1136/bmjopen-2016-013191 PMC5073537 27697882

[41] Ebrahimpour-koujan S, Saneei P, Larijani B, Esmaillzadeh A (2020) Consumption of sugar sweetened beverages and dietary fructose in relation to risk of gout and hyperuricemia: a systematic review and meta-analysis. Crit Rev Food Sci Nutr 60(1):1–10. 10.1080/10408398.2018.1503155 30277800

[42] Ebrahimpour-koujan S, Saneei P, Larijani B, Esmaillzadeh A (2021) Consumption of sugar‐sweetened beverages and serum uric acid concentrations: a systematic review and meta‐analysis. J Hum Nutr Diet 34(2):305–313. 10.1111/jhn.12796 32683776

[43] Ayoub-Charette S, Liu Q, Khan TA, Au-Yeung F, Blanco Mejia S, De Souza RJ et al (2019) Important food sources of fructose-containing sugars and incident gout: a systematic review and meta-analysis of prospective cohort studies. BMJ Open 9(5):e024171. 10.1136/bmjopen-2018-024171 PMC6502023 31061018

[44] Singh JA, Reddy SG, Kundukulam J (2011) Risk factors for gout and prevention: a systematic review of the literature. Curr Opin Rheumatol 23(2):192–202. 10.1097/BOR.0b013e3283438e13 PMC4104583 21285714

[45] Lu Y, Wang Y, Huang R, Pan H, Xie Z, Wen C et al (2025) Sugar-sweetened beverages and the risk of hyperuricemia and gout: a meta-analysis. Front Nutr 12:1669129. 10.3389/fnut.2025.1669129 PMC12580599 41190154

[46] Tan H, Tang P, Chai H, Ma W, Cao Y, Lin B et al (2025) Beverage consumption on bone and joint disorders: an umbrella review. J Glob Health 15:04222. 10.7189/jogh.15.04222 PMC12319397 40755011

[47] D’Elia L, Zarrella AF, Pizzulo IL, Attanasio A, Rendina D, Sievenpiper JL (2025) 100% fruit juice intake, glucose profile, and uric acid: a systematic review. J Med Food 28(8):743–56. 10.1089/jmf.2024.0139 40512554

[48] Yu SJ, Lee HS, Gung HJ, Kim JS, Kim KB, Kwon YH et al (2022) Efficacy of a restrictive diet in irritable bowel syndrome: a systematic review and network meta-analysis. Korean J Gastroenterol 80(1):6–16. 10.4166/kjg.2022.014 PMC12285310 35879058

[49] Black CJ, Staudacher HM, Ford AC (2022) Efficacy of a low FODMAP diet in irritable bowel syndrome: systematic review and network meta-analysis. Gut 71(6):1117–1126. 10.1136/gutjnl-2021-325214 34376515

[50] Dionne J, Ford AC, Yuan Y, Chey WD, Lacy BE, Saito YA et al (2018) A systematic review and meta-analysis evaluating the efficacy of a gluten-free diet and a low FODMAPS diet in treating symptoms of irritable bowel syndrome. Am J Gastroenterol 113(9):1290–1300. 10.1038/s41395-018-0195-4 30046155

[51] Hahn J, Choi J, Chang MJ (2021) Effect of low FODMAPs diet on irritable bowel syndromes: a systematic review and meta-analysis of clinical trials. Nutrients 13(7):2460. 10.3390/nu13072460 PMC8308820 34371973

[52] Khademi Z, Milajerdi A, Larijani B, Esmaillzadeh A (2021) Dietary intake of total carbohydrates, sugar and sugar-sweetened beverages, and risk of inflammatory bowel disease: a systematic review and meta-analysis of prospective cohort studies. Front Nutr 8:707795. 10.3389/fnut.2021.707795 PMC8517080 34660658

[53] Marsh A, Eslick EM, Eslick GD (2016) Does a diet low in FODMAPs reduce symptoms associated with functional gastrointestinal disorders? A comprehensive systematic review and meta-analysis. Eur J Nutr 55(3):897–906. 10.1007/s00394-015-0922-1 25982757

[54] Rao SSC, Yu S, Fedewa A (2015) Systematic review: dietary fibre and FODMAP -restricted diet in the management of constipation and irritable bowel syndrome. Aliment Pharmacol Ther 41(12):1256–1270. 10.1111/apt.13167 25903636

[55] So D, Loughman A, Staudacher HM (2022) Effects of a low FODMAP diet on the colonic microbiome in irritable bowel syndrome: a systematic review with meta-analysis. Am J Clin Nutr 116(4):943–952. 10.1093/ajcn/nqac176 PMC9535515 35728042

[56] Spooren CEGM, Pierik MJ, Zeegers MP, Feskens EJM, Masclee AAM, Jonkers DMAE (2013) Review article: the association of diet with onset and relapse in patients with inflammatory bowel disease. Aliment Pharmacol Ther 38(10):1172–1187. 10.1111/apt.12501 24118051

[57] Su H, Li YT, Heitkemper MM, Zia J (2019) Effects of low-FODMAPS diet on irritable bowel syndrome symptoms and gut microbiome. Gastroenterol Nurs 42(2):150–158. 10.1097/SGA.0000000000000428 30946302

[58] Stróżyk A, Horvath A, Szajewska H (2022) FODMAP dietary restrictions in the management of children with functional abdominal pain disorders: a systematic review. Neurogastroenterology Motil 34(10):e14345. 10.1111/nmo.14345 35231146

[59] Turco R, Salvatore S, Miele E, Romano C, Marseglia GL, Staiano A (2018) Does a low FODMAPs diet reduce symptoms of functional abdominal pain disorders? a systematic review in adult and paediatric population, on behalf of Italian Society of Pediatrics. Ital J Pediatr 44(1):53. 10.1186/s13052-018-0495-8 PMC5952847 29764491

[60] Varjú P, Farkas N, Hegyi P, Garami A, Szabó I, Illés A et al (2017) Low fermentable oligosaccharides, disaccharides, monosaccharides and polyols (FODMAP) diet improves symptoms in adults suffering from irritable bowel syndrome (IBS) compared to standard IBS diet: a meta-analysis of clinical studies. PLoS One 12(8):e0182942. 10.1371/journal.pone.0182942 PMC5555627 28806407

[61] Van Lanen AS, De Bree A, Greyling A (2021) Correction to: efficacy of a low-FODMAP diet in adult irritable bowel syndrome: a systematic review and meta-analysis. Eur J Nutr 60(6):3523–3523. 10.1007/s00394-021-02620-1 PMC8354870 34181070

[62] Lusetti F, Schiepatti A, Scalvini D, Maimaris S, Biagi F (2024) Efficacy of a low-FODMAP diet for coeliac patients with persistent IBS-like symptoms despite a gluten-free diet: a systematic review. Nutrients 16(7):1094. 10.3390/nu16071094 PMC11013587 38613127

[63] Christensen C, Knudsen A, Arnesen EK, Hatlebakk JG, Sletten IS, Fadnes LT (2024) Diet, food, and nutritional exposures and inflammatory bowel disease or progression of disease: an umbrella review. Adv Nutr 15(5):100219. 10.1016/j.advnut.2024.100219 PMC11063602 38599319

[64] Pouladi A, Arabpour E, Bahrami O, Sadeghi A, Mozafari Komesh Tape P, Abdehagh M et al (2025) Impacts of the long-term low‐FODMAP diet in patients with irritable bowel syndrome: a systematic review and meta‐analysis. J Hum Nutr Diet 38(4):e70105. 10.1111/jhn.70105 40735813

[65] Maniyar I, Jajoo A, Brewer GG, Iyengar P, Nguyen A, Fasulo C et al (2025) Dietary sugar and sweetened beverage intake increases inflammatory bowel disease risk: a systematic review and meta-analysis. UEG J 13(8):1379–1409. 10.1002/ueg2.70047 PMC12529010 40532067

[66] Kuźmin L, Kubiak K, Lange E (2025) Efficacy of a Low-FODMAP diet on the severity of gastrointestinal symptoms and quality of life in the treatment of gastrointestinal disorders—A. Syst Rev Randomized Controlled Trials Nutrients 17(12):2045. 10.3390/nu17122045 PMC12196412 40573159

[67] Singh R, Salem A, Nanavati J, Mullin GE (2018) The role of diet in the treatment of irritable bowel syndrome: a systematic review. Gastroenterol Clin North Am 47(1):107–137. 10.1016/j.gtc.2017.10.003 PubMed PMID: 29413008 29413008

[68] Chen H, Wang J, Li Z, Lam CWK, Xiao Y, Wu Q et al (2019) Consumption of sugar-sweetened beverages has a dose-dependent effect on the risk of non-alcoholic fatty liver disease: an updated systematic review and dose-response meta-analysis. Int J Environ Res Public Health 16(12):12. 10.3390/ijerph16122192 PubMed PMID: 31234281; PubMed Central PMCID: PMC6617076 PMC6617076 31234281

[69] Chiu S, Sievenpiper JL, de Souza RJ, Cozma AI, Mirrahimi A, Carleton AJ et al (2014) Effect of fructose on markers of non-alcoholic fatty liver disease (NAFLD): a systematic review and meta-analysis of controlled feeding trials. Eur J Clin Nutr. 10.1038/ejcn.2014.8 PMC3975811 24569542

[70] Chung M, Ma J, Patel K, Berger S, Lau J, Lichtenstein AH (2014) Fructose, high-fructose corn syrup, sucrose, and nonalcoholic fatty liver disease or indexes of liver health: a systematic review and meta-analysis1234. Am J Clin Nutr 100(3):3. 10.3945/ajcn.114.086314 PubMed PMID: 25099546; PubMed Central PMCID: PMC4135494 PMC4135494 25099546

[71] Lee D, Chiavaroli L, Ayoub-Charette S, Khan TA, Zurbau A, Au-Yeung F et al (2022) Important food sources of fructose-containing sugars and non-alcoholic fatty liver disease: a systematic review and meta-analysis of controlled trials. Nutrients. 10.3390/nu14142846 PMC9325155 35889803

[72] Wijarnpreecha K, Thongprayoon C, Edmonds PJ, Cheungpasitporn W (2016) Associations of sugar- and artificially sweetened soda with nonalcoholic fatty liver disease: a systematic review and meta-analysis. QJM 109(7):461–6. 10.1093/qjmed/hcv172 26385233

[73] Asgari-Taee F, Zerafati-Shoae N, Dehghani M, Sadeghi M, Baradaran HR, Jazayeri S (2019) Association of sugar sweetened beverages consumption with non-alcoholic fatty liver disease: a systematic review and meta-analysis. Eur J Nutr 58(5):1759–69. 10.1007/s00394-018-1711-4 29761318

[74] Qin P, Li Q, Zhao Y, Chen Q, Sun X, Liu Y et al (2020) Sugar and artificially sweetened beverages and risk of obesity, type 2 diabetes mellitus, hypertension, and all-cause mortality: a dose–response meta-analysis of prospective cohort studies. Eur J Epidemiol 35(7):655–671. 10.1007/s10654-020-00655-y 32529512

[75] Ruanpeng D, Thongprayoon C, Cheungpasitporn W, Harindhanavudhi T (2017) Sugar and artificially sweetened beverages linked to obesity: a systematic review and meta-analysis. QJM: Int J Med 110(8):513–520. 10.1093/qjmed/hcx068 28402535

[76] Trumbo PR, Rivers CR (2014) Systematic review of the evidence for an association between sugar-sweetened beverage consumption and risk of obesity. Nutr Rev 72(9):566–574. 10.1111/nure.12128 25091794

[77] Bucher Della Torre S, Keller A, Laure Depeyre J, Kruseman M (2016) Sugar-sweetened beverages and obesity risk in children and adolescents: a systematic analysis on how methodological quality may influence conclusions. J Acad Nutr Diet 116(4):638–59. 10.1016/j.jand.2015.05.020 26194333

[78] Gibson S (2008) Sugar-sweetened soft drinks and obesity: a systematic review of the evidence from observational studies and interventions. Nutr Res Rev 21(2):134–147. 10.1017/S0954422408110976 19087367

[79] Nissensohn M, Fuentes Lugo D, Serra-Majem L (2018) Sugar-sweetened beverage consumption and obesity in children’s meta-analyses: reaching wrong answers for right questions. Nutr Hosp. 10.20960/nh.1492 29756984

[80] Luger M, Lafontan M, Bes-Rastrollo M, Winzer E, Yumuk V, Farpour-Lambert N (2017) Sugar-sweetened beverages and weight gain in children and adults: a systematic review from 2013 to 2015 and a comparison with previous studies. Obes Facts 10(6):674–693. 10.1159/000484566 PMC5836186 29237159

[81] Malik VS, Schulze MB, Hu FB (2006) Intake of sugar-sweetened beverages and weight gain: a systematic review. Am J Clin Nutr 84(2):2. 10.1093/ajcn/84.1.274 PubMed PMID: 16895873; PubMed Central PMCID: PMC3210834 PMC3210834 16895873

[82] Malik VS, Pan A, Willett WC, Hu FB (2013) Sugar-sweetened beverages and weight gain in children and adults: a systematic review and meta-analysis. Am J Clin Nutr. 10.3945/ajcn.113.058362 PMC3778861 23966427

[83] Neelakantan N, Park SH, Chen GC, Van Dam RM (2021) Sugar-sweetened beverage consumption, weight gain, and risk of type 2 diabetes and cardiovascular diseases in Asia: a systematic review. Nutr Rev 80(1):50–67. 10.1093/nutrit/nuab010 33855443

[84] Santos LP, Gigante DP, Delpino FM, Maciel AP, Bielemann RM (2022) Sugar sweetened beverages intake and risk of obesity and cardiometabolic diseases in longitudinal studies: a systematic review and meta-analysis with 1.5 million individuals. Clin Nutr ESPEN 51:128–142. 10.1016/j.clnesp.2022.08.021 36184197

[85] Abbasalizad Farhangi M, Mohammadi Tofigh A, Jahangiri L, Nikniaz Z, Nikniaz L (2022) Sugar-sweetened beverages intake and the risk of obesity in children: an updated systematic review and dose–response meta‐analysis. Pediatr Obes 17(8):e12914. 10.1111/ijpo.12914 35466543

[86] Ardeshirlarijani E, Jalilpiran Y, Daneshzad E, Larijani B, Namazi N, Azadbakht L (2021) Association between sugar-sweetened beverages and waist circumference in adult populations: a meta-analysis of prospective cohort studies. Clin Nutr ESPEN 41:118–125. 10.1016/j.clnesp.2020.10.014 33487253

[87] Café ACC, Lopes CADO, Novais RLR, Bila WC, Silva DKD, Romano MCC, Consumo de bebidas açucaradas, leite e sua associação com o índice de massa corporal na adolescência: uma revisão sistemática et al (2018) Rev paul pediatr 36(1):91–99. 10.1590/1984-0462. (**/;2018;36;1;00010**) PMC5849383 29513855

[88] Forshee RA, Anderson PA, Storey ML (2008) Sugar-sweetened beverages and body mass index in children and adolescents: a meta-analysis. Am J Clin Nutr 87(6):1662–1671. 10.1093/ajcn/87.6.1662 18541554

[89] Frantsve-Hawley J, Bader JD, Welsh JA, Wright JT (2017) A systematic review of the association between consumption of sugar‐containing beverages and excess weight gain among children under age 12. J Public Health Dent. 10.1111/jphd.12222 28556932

[90] Eugenia Pérez-Morales MBG y AJC (2013) Ingesta de bebidas azucaradas antes de los seis años y peso o IMC en los niños/adolescentes: revisión sistemática. Nutr Hosp (1):47–51. 10.3305/nh.2013.28.1.6247

[91] Rousham EK, Goudet S, Markey O, Griffiths P, Boxer B, Carroll C et al (2022) Unhealthy food and beverage consumption in children and risk of overweight and obesity: a systematic review and meta-analysis. Adv Nutr 13(5):1669–1696. 10.1093/advances/nmac032 PMC9526862 35362512

[92] Jakobsen DD, Brader L, Bruun JM (2023) Effects of foods, beverages and macronutrients on BMI z-score and body composition in children and adolescents: a systematic review and meta-analysis of randomized controlled trials. Eur J Nutr 62(1):1–15. 10.1007/s00394-022-02966-0 35902429

[93] Duncanson K, Shrewsbury V, Burrows T, Chai LK, Ashton L, Taylor R et al (2021) Impact of weight management nutrition interventions on dietary outcomes in children and adolescents with overweight or obesity: a systematic review with meta-analysis. J Hum Nutr Diet 34(1):147–77. 10.1111/jhn.12831 33283363

[94] English LK, Obbagy JE, Wong YP, Butte NF, Dewey KG, Fox MK et al (2019) Types and amounts of complementary foods and beverages consumed and growth, size, and body composition: a systematic review. Am J Clin Nutr 109:956S–977S. 10.1093/ajcn/nqy281 30982866

[95] Fogelholm M, Anderssen S, Gunnarsdottir I, Lahti-Koski M (2012) Dietary macronutrients and food consumption as determinants of long-term weight change in adult populations: a systematic literature review. Food Nutr Res 56(1):19103. 10.3402/fnr.v56i0.19103 PMC3418611 22893781

[96] Schlesinger S, Neuenschwander M, Schwedhelm C, Hoffmann G, Bechthold A, Boeing H et al (2019) Food groups and risk of overweight, obesity, and weight gain: a systematic review and dose-response meta-analysis of prospective studies. Adv Nutr 10(2):205–218. 10.1093/advances/nmy092 PMC6416048 30801613

[97] Schneider BC, Dumith SC, Orlandi SP, Assunção MCF (2017) Diet and body fat in adolescence and early adulthood: a systematic review of longitudinal studies. Ciênc saúde coletiva 22(5):1539–1552. 10.1590/1413-81232017225.13972015 28538925

[98] Te Morenga L, Mallard S, Mann J (2012) Dietary sugars and body weight: systematic review and meta-analyses of randomised controlled trials and cohort studies. BMJ 346(jan15 3):e7492–e7492. 10.1136/bmj.e7492 23321486

[99] Yang WY, Williams LT, Collins C, Siew Swee CW (2012) The relationship between dietary patterns and overweight and obesity in children of Asian developing countries: a systematic review: JBI database of systematic reviews and implementation reports. 10(58):4568–4599. 10.11124/jbisrir-2012-407 27820524

[100] Ma J, Karlsen MC, Chung M, Jacques PF, Saltzman E, Smith CE et al (2016) Potential link between excess added sugar intake and ectopic fat: a systematic review of randomized controlled trials. Nutr Rev 74(1):18–32. 10.1093/nutrit/nuv047 PMC4859325 26518034

[101] Poorolajal J, Sahraei F, Mohamdadi Y, Doosti-Irani A, Moradi L (2020) Behavioral factors influencing childhood obesity: a systematic review and meta-analysis. Obes Res Clin Pract 14(2):109–118. 10.1016/j.orcp.2020.03.002 32199860

[102] Liberali R, Del Castanhel F, Kupek E, Assis MAAD (2021) Latent class analysis of lifestyle risk factors and association with overweight and/or obesity in children and adolescents: systematic review. Child Obes 17(1):2–15. 10.1089/chi.2020.0115 33306451

[103] Kang YJ, Wang HW, Cheon SY, Lee HJ, Hwang KM, Yoon HS (2016) Associations of obesity and dyslipidemia with intake of sodium, fat, and sugar among koreans: a qualitative systematic review. Clin Nutr Res 5(4):290. 10.7762/cnr.2016.5.4.290 PMC5093226 27812518

[104] Zhang X, Li X, Liu L, Hong F, Zhao H, Chen L et al (2021) Dose–response association between sugar- and artificially sweetened beverage consumption and the risk of metabolic syndrome: a meta-analysis of population-based epidemiological studies. Public Health Nutr 24(12):3892–3904. 10.1017/S1368980020003614 PMC10195306 33109289

[105] Narain A, Kwok CS, Mamas MA (2017) Soft drink intake and the risk of metabolic syndrome: a systematic review and meta-analysis. Int J Clin Pract 71(2):e12927. 10.1111/ijcp.12927 28074617

[106] Kelishadi R, Mansourian M, Heidari-Beni M (2014) Association of fructose consumption and components of metabolic syndrome in human studies: a systematic review and meta-analysis. Nutrition 30(5):503–510. 10.1016/j.nut.2013.08.014 24698343

[107] Sievenpiper JL, De Souza RJ, Mirrahimi A, Yu ME, Carleton AJ, Beyene J et al (2012) Effect of fructose on body weight in controlled feeding trials: a systematic review and meta-analysis. Ann Intern Med 156(4):291–304. 10.7326/0003-4819-156-4-201202210-00007 22351714

[108] Semnani-Azad Z, Khan TA, Blanco Mejia S, De Souza RJ, Leiter LA, Kendall CWC et al (2020) Association of major food sources of fructose-containing sugars with incident metabolic syndrome: a systematic review and meta-analysis. JAMA Netw Open 3(7):e209993. 10.1001/jamanetworkopen.2020.9993 PMC7348689 32644139

[109] Liberali R, Kupek E, Assis MAAD (2020) Dietary patterns and childhood obesity risk: a systematic review. Child Obes 16(2):70–85. 10.1089/chi.2019.0059 31742427

[110] Thornhill K, Charlton K, Probst Y, Neale E (2019) Does an increased intake of added sugar affect appetite in overweight or obese adults, when compared with lower intakes? A systematic review of the literature. Br J Nutr 121(2):232–240. 10.1017/S0007114518003239 30489234

[111] Keller A, Bucher Della Torre S (2015) Sugar-sweetened beverages and obesity among children and adolescents: a review of systematic literature reviews. Child Obes 11(4):338–346. 10.1089/chi.2014.0117 PMC4529053 26258560

[112] Sanders LM, Dicklin MR, Zhu Y, Maki KC (2023) The Impact of Ready-to-eat cereal intake on body weight and body composition in children and adolescents: a systematic review of observational studies and controlled trials. Adv Nutr 14(1):161–172. 10.1016/j.advnut.2022.11.003 PMC10102992 36811587

[113] Patro-Gołąb B, Zalewski BM, Kołodziej M, Kouwenhoven S, Poston L, Godfrey KM et al (2016) Nutritional interventions or exposures in infants and children aged up to 3 years and their effects on subsequent risk of overweight, obesity and body fat: a systematic review of systematic reviews. Obes Rev 17(12):1245–1257. 10.1111/obr.12476 PMC5325317 27749991

[114] Nguyen M, Jarvis SE, Chiavaroli L, Mejia SB, Zurbau A, Khan TA et al (2024) Consumption of 100% fruit juice and body weight in children and adults: a systematic review and meta-analysis. JAMA Pediatr 178(3):237. 10.1001/jamapediatrics.2023.6124 PMC10792499 38227336

[115] Kristoffersen E, Hjort SL, Thomassen LM, Arjmand EJ, Perillo M, Balakrishna R et al (2025) Umbrella review of systematic reviews and meta-analyses on the consumption of different food groups and the risk of overweight and obesity. Nutrients 17(4):662. 10.3390/nu17040662 PMC11857968 40004990

[116] Giahi L, Mohammadmoradi S, Javidan A, Sadeghi MR (2015) Nutritional modifications in male infertility: a systematic review covering 2 decades. Nutr Rev. 10.1093/nutrit/nuv059 PMC4892303 26705308

[117] Salas-Huetos A, Bulló M, Salas-Salvadó J (2017) Dietary patterns, foods and nutrients in male fertility parameters and fecundability: a systematic review of observational studies. Hum Reprod Update 23(4):4. 10.1093/humupd/dmx006 28333357

[118] Mayer-Davis E, Leidy H, Mattes R, Naimi T, Novotny R, Schneeman B et al Beverage consumption during pregnancy and birth weight: a systematic review [Internet]. Alexandria (VA): USDA Nutrition evidence systematic review; 2020 [cited 2024 Nov 8]. (USDA Nutrition Evidence Systematic Reviews). Available from: http://www.ncbi.nlm.nih.gov/books/NBK578891/ PubMed PMID: 35349234 35349234

[119] Chia AR, Chen LW, Lai JS, Wong CH, Neelakantan N, Van Dam RM et al (2019) Maternal dietary patterns and birth outcomes: a systematic review and meta-analysis. Adv Nutr 10(4):685–695. 10.1093/advances/nmy123 PMC6628847 31041446

[120] Litvak J, Parekh N, Deierlein A (2020) Prenatal dietary exposures and offspring body size from 6 months to 18 years: a systematic review. Pediatr Perinat Epid 34(2):2. 10.1111/ppe.12629 32011754

[121] Gupta A, Singh A, Fernando RL, Dharmage SC, Lodge CJ, Waidyatillake NT (2022) The association between sugar intake during pregnancy and allergies in offspring: a systematic review and a meta-analysis of cohort studies. Nutr Rev 80(4):904–918. 10.1093/nutrit/nuab052 PubMed PMID: 34432049 34432049

[122] Ibrahim MO, Abuhijleh H, Tayyem R (2023) What Dietary Patterns and Nutrients Are Associated With Pancreatic Cancer? Literature Review. Cancer Manag Res 15:17–30 doi:10.2147/CMAR.S390228 PubMed PMID: 36643074; PubMed Central PMCID: PMC9832506 10.2147/CMAR.S390228 PMC9832506 36643074

[123] Kazemi A, Barati-Boldaji R, Soltani S, Mohammadipoor N, Esmaeilinezhad Z, Clark CCT et al (2020) Intake of Various Food Groups and Risk of Breast Cancer: A Systematic Review and Dose-Response Meta-Analysis of Prospective Studies. Adv Nutr 12(3):3. 10.1093/advances/nmaa147 PubMed PMID: 33271590; PubMed Central PMCID: PMC8166564 PMC8166564 33271590

[124] Aune D, Chan DSM, Vieira AR, Navarro Rosenblatt DA, Vieira R, Greenwood DC et al (2012) Dietary fructose, carbohydrates, glycemic indices and pancreatic cancer risk: a systematic review and meta-analysis of cohort studies. Ann Oncol 23(10):10. 10.1093/annonc/mds076 22539563

[125] Carroll KL, Frugé AD, Heslin MJ, Lipke EA, Greene MW (2022) Diet as a risk factor for early-onset colorectal adenoma and carcinoma: a systematic review. Front Nutr 9:896330. 10.3389/fnut.2022.896330 PubMed PMID: 35757246; PubMed Central PMCID: PMC9218641 PMC9218641 35757246

[126] Conigliaro T, Boyce LM, Lopez CA, Tonorezos ES (2020) Food intake during cancer therapy: a systematic review. Am J Clin Oncol 43(11):11. 101097/COC.0000000000000749 PubMed PMID: 32889891; PubMed Central PMCID: PMC7584741 10.1097/COC.0000000000000749 PMC7584741 32889891

[127] Azeem S, Gillani SW, Siddiqui A, Jandrajupalli SB, Poh V, Sulaiman SAS (2015) Diet and colorectal cancer risk in Asia - a systematic Review. Asian Pac J Cancer Prev 16(13):13. 10.7314/APJCP.2015.16.13.5389 26225683

[128] Makarem N, Bandera EV, Nicholson JM, Parekh N (2018) Consumption of sugars, sugary foods, and sugary beverages in relation to cancer risk: a systematic review of longitudinal studies. Annu Rev Nutr 38:17–39. 10.1146/annurev-nutr-082117-051805 PubMed PMID: 29801420 29801420

[129] Li Y, Guo L, liangzi, He K, Huang C, Tang S (2021) Consumption of sugar-sweetened beverages and fruit juice and human cancer: a systematic review and dose-response meta-analysis of observational studies. J Cancer 12(10):10. 10.7150/jca.51322 PubMed PMID: 33854607; PubMed Central PMCID: PMC8040874 PMC8040874 33854607

[130] Llaha F, Gil-Lespinard M, Unal P, de Villasante I, Castañeda J, Zamora-Ros R (2021) Consumption of sweet beverages and cancer risk. a systematic review and meta-analysis of observational studies. Nutrients 13(2):2. 10.3390/nu13020516 PubMed PMID: 33557387; PubMed Central PMCID: PMC7915548 PMC7915548 33557387

[131] Ghosh NR, Jones LA (2022) Dietary risk factors for esophageal cancer based on world health organization regions. Nutrition 95:111552. 10.1016/j.nut.2021.111552 34999383

[132] Fretwell A, Louca P, Cohoon G, Sakellaropoulou A, Henriques Caetano MDP, Koullapis A et al (2025) Still too little evidence: the role of diet in colorectal cancer survivorship - a systematic review. Crit Rev Food Sci Nutr 65(16):3173–3185. 10.1080/10408398.2024.2360068 38860747

[133] Li N, Guo X, Sun C, Lowe S, Su W, Song Q et al (2022) Dietary carbohydrate intake is associated with a lower risk of breast cancer: a meta-analysis of cohort studies. Nutr Res 100:70–92. 10.1016/j.nutres.2022.01.004 PubMed PMID: 35220083 35220083

[134] Schwingshackl L, Schwedhelm C, Hoffmann G, Knüppel S, Laure Preterre A, Iqbal K et al (2018) Food groups and risk of colorectal cancer. Int J Cancer 142(9):1748–1758. 10.1002/ijc.31198 PubMed PMID: 29210053 29210053

[135] Bartrina JA, Rodrigo CP (2013) The relationship between sucrose consumption and cancer; a review of the evidence. Nutrición hospitalaria 28(Suppl 4):95–105 10.3305/nh.2013.28.sup4.6802 23834098

[136] Bergwall S, Johansson A, Sonestedt E, Acosta S (2022) High versus low-added sugar consumption for the primary prevention of cardiovascular disease. Cochrane Heart Group editor Cochrane Database Syst Reviews 2022(1):1. 10.1002/14651858.CD013320.pub2 PMC8730703 34986271

[137] Mayer-Davis E, Leidy H, Mattes R, Naimi T, Novotny R, Schneeman B (2020) Added Sugars consumption and risk of cardiovascular disease: a systematic review. U.S. Department of agriculture, food and nutrition service, center for nutrition policy and promotion, nutrition evidence systematic review. 10.52570/NESR.DGAC2020.SR0404 35353466

[138] Sun Q, Yang Y, Wang X, Yang R, Li X (2022) The association between sugar-sweetened beverages and cognitive function in middle-aged and older people: a meta-analysis. J Prev Alzheimers Dis 9(2):2. 10.14283/jpad.2021.71 35543006

[139] Gibson S, Gunn P, Wittekind A, Cottrell R (2013) The effects of sucrose on metabolic health: a systematic review of human intervention studies in healthy adults. Crit Rev Food Sci Nutr 53(6):6. 10.1080/10408398.2012.691574 PMC3630450 23627502

[140] Ahmed A, Tul-Noor Z, Lee D, Bajwah S, Ahmed Z, Zafar S et al (2023) Effect of honey on cardiometabolic risk factors: a systematic review and meta-analysis. Nutr Rev 81(7):7. 10.1093/nutrit/nuac086 PMC10251304 36379223

[141] Schwingshackl L, Schwedhelm C, Hoffmann G, Knüppel S, Iqbal K, Andriolo V et al (2017) Food groups and risk of hypertension: a systematic review and dose-response meta-analysis of prospective studies. Adv Nutr 8(6):6. 10.3945/an.117.017178 PMC5683007 29141965

[142] Bhagavathula AS, Rahmani J, Vidyasagar K, Tesfaye W, Khubchandani J (2022) Sweetened beverage consumption and risk of cardiovascular mortality: a systematic review and meta-analysis. Diabetes Metabolic Syndrome: Clin Res Reviews 16(4):4. 10.1016/j.dsx.2022.102462 35325785

[143] Alhabeeb H, Sohouli MH, Lari A, Fatahi S, Shidfar F, Alomar O et al (2022) Impact of orange juice consumption on cardiovascular disease risk factors: a systematic review and meta-analysis of randomized-controlled trials. Crit Rev Food Sci Nutr 62(12):12. 10.1080/10408398.2020.1865263 33350317

[144] Khan TA, Tayyiba M, Agarwal A, Mejia SB, De Souza RJ, Wolever TMS et al (2019) Relation of total sugars, sucrose, fructose, and added sugars with the risk of cardiovascular disease. Mayo Clin Proc 94(12):12. 10.1016/j.mayocp.2019.05.034 31806098

[145] Huang C, Huang J, Tian Y, Yang X, Gu D (2014) Sugar sweetened beverages consumption and risk of coronary heart disease: a meta-analysis of prospective studies. Atherosclerosis 234(1):1. 10.1016/j.atherosclerosis.2014.01.037 24583500

[146] Liu Q, Ayoub-Charette S, Khan TA, Au‐Yeung F, Blanco Mejia S, De Souza RJ et al (2019) Important food sources of fructose‐containing sugars and incident hypertension: a systematic review and dose‐response meta‐analysis of prospective cohort studies. JAHA 8(24):24. 10.1161/JAHA.118.010977 PMC6951071 31826724

[147] Khanji MY, Van Waardhuizen CN, Bicalho VVS, Ferket BS, Hunink MGM, Petersen SE (2018) Lifestyle advice and interventions for cardiovascular risk reduction: A systematic review of guidelines. Int J Cardiol 263:142–151. 10.1016/j.ijcard.2018.02.094 29754910

[148] Moosavian SP, Maharat M, Chambari M, Moradi F, Rahimlou M (2022) Effects of tart cherry juice consumption on cardio-metabolic risk factors: a systematic review and meta-analysis of randomized-controlled trials. Complement Ther Med 71:102883. 10.1016/j.ctim.2022.102883 36038032

[149] Yin J, Zhu Y, Malik V, Li X, Peng X, Zhang FF et al (2021) Intake of sugar-sweetened and low-calorie sweetened beverages and risk of cardiovascular disease: a meta-analysis and systematic review. Adv Nutr 12(1):1. 10.1093/advances/nmaa084 PMC7850046 32696948

[150] Schwingshackl L, Neuenschwander M, Hoffmann G, Buyken AE, Schlesinger S (2020) Dietary sugars and cardiometabolic risk factors: a network meta-analysis on isocaloric substitution interventions. Am J Clin Nutr 111(1):1. 10.1093/ajcn/nqz273 31711109

[151] Hoare E, Varsamis P, Owen N, Dunstan DW, Jennings GL, Kingwell BA (2017) Sugar- and intense-sweetened drinks in australia: a systematic review on cardiometabolic risk. Nutrients 9(10):10. 10.3390/nu9101075 PubMed PMID: 28956823; PubMed Central PMCID: PMC5691692 PMC5691692 28956823

[152] Te Morenga LA, Howatson AJ, Jones RM, Mann J (2014) Dietary sugars and cardiometabolic risk: systematic review and meta-analyses of randomized controlled trials of the effects on blood pressure and lipids. Am J Clin Nutr 100(1):1. 10.3945/ajcn.113.081521 PubMed PMID: 24808490 24808490

[153] Farhangi MA, Nikniaz L, Khodarahmi M (2020) Sugar-sweetened beverages increases the risk of hypertension among children and adolescence: a systematic review and dose–response meta-analysis. J Transl Med 18:344. 10.1186/s12967-020-02511-9 PubMed PMID: 32891165; PubMed Central PMCID: PMC7487688 PMC7487688 32891165

[154] Deng C, Lu Q, Gong B, Li L, Chang L, Fu L et al (2018) Stroke and food groups: an overview of systematic reviews and meta-analyses. Public Health Nutr 21(4):4. 10.1017/S1368980017003093 PMC10260832 29143697

[155] Jayalath VH, Sievenpiper JL, De Souza RJ, Ha V, Mirrahimi A, Santaren ID et al (2014) Total fructose intake and risk of hypertension: a systematic review and meta-analysis of prospective cohorts. J Am Coll Nutr 33(4):4. 10.1080/07315724.2014.916237 PMC4261182 25144126

[156] Fattore E, Botta F, Bosetti C (2021) Effect of fructose instead of glucose or sucrose on cardiometabolic markers: a systematic review and meta-analysis of isoenergetic intervention trials. Nutr Rev 79(2):2. 10.1093/nutrit/nuaa077 33029629

[157] Kim Y, Je Y (2016) Prospective association of sugar-sweetened and artificially sweetened beverage intake with risk of hypertension. Arch Cardiovasc Dis. 10.1016/j.acvd.2015.10.005 26869455

[158] Jayalath VH, De Souza RJ, Ha V, Mirrahimi A, Blanco-Mejia S, Di Buono M et al (2015) Sugar-sweetened beverage consumption and incident hypertension: a systematic review and meta-analysis of prospective cohorts. Am J Clin Nutr 102(4):4. 10.3945/ajcn.115.107243 26269365

[159] Xi B, Huang Y, Reilly KH, Li S, Zheng R, Barrio-Lopez MT et al (2015) Sugar-sweetened beverages and risk of hypertension and CVD: a dose–response meta-analysis. Br J Nutr 113(5):5. 10.1017/S0007114514004383 25735740

[160] Malik AH, Akram Y, Shetty S, Malik SS, Yanchou Njike V (2014) Impact of sugar-sweetened beverages on blood pressure. Am J Cardiol 113(9):9. 10.1016/j.amjcard.2014.01.437 24630785

[161] Cheungpasitporn W, Thongprayoon C, Edmonds PJ, Srivali N, Ungprasert P, Kittanamongkolchai W et al (2015) Sugar and artificially sweetened soda consumption linked to hypertension: A systematic review and meta-analysis. Clin Exp Hypertens 37(7):7. 10.3109/10641963.2015.1026044 26114357

[162] Keller A, Heitmann BL, Olsen N (2015) Sugar-sweetened beverages, vascular risk factors and events: a systematic literature review. Public Health Nutr 18(7):7. 10.1017/S1368980014002122 PMC10271571 25321082

[163] Song S, Shim JE, Song Y (2022) Association of added sugar intake with all-cause and cardiovascular disease mortality: a systematic review of cohort studies. Nutr Res Pract 16(Suppl 1):Suppl1. 10.4162/nrp.2022.16.S1.S21 PMC9127522 35651837

[164] Auerbach BJ, Dibey S, Vallila-Buchman P, Kratz M, Krieger J (2018) Review of 100% fruit juice and chronic health conditions: implications for sugar-sweetened beverage policy. Adv Nutr 9(2):78–85. 10.1093/advances/nmx006 PMC5916434 29659683

[165] Kazemi A, Soltani S, Mokhtari Z, Khan T, Golzarand M, Hosseini E et al (2023) The relationship between major food sources of fructose and cardiovascular disease, cancer, and all-cause mortality: a systematic review and dose-response meta-analysis of cohort studies. Crit Rev Food Sci Nutr 63(20):20. 10.1080/10408398.2021.2000361 34847334

[166] Wang Y, Zhao R, Wang B, Zhao C, Zhu B, Tian X (2022) The dose-response associations of sugar-sweetened beverage intake with the risk of stroke, depression, cancer, and cause-specific mortality: a systematic review and meta-analysis of prospective studies. Nutrients 14(4):4. 10.3390/nu14040777 PubMed PMID: 35215425; PubMed Central PMCID: PMC8875574 PMC8875574 35215425

[167] Micha R, Shulkin ML, Peñalvo JL, Khatibzadeh S, Singh GM, Rao M et al (2017) Etiologic effects and optimal intakes of foods and nutrients for risk of cardiovascular diseases and diabetes: systematic reviews and meta-analyses from the Nutrition and Chronic Diseases Expert Group (NutriCoDE). PLoS One. 10.1371/journal.pone.0175149 PMC5407851 28448503

[168] Sonestedt E, Øverby N, Laaksonen DE, Eva Birgisdottir B (2012) Does high sugar consumption exacerbate cardiometabolic risk factors and increase the risk of type 2 diabetes and cardiovascular disease? Food Nutr Res 56(1):1. 10.3402/fnr.v56i0.19104 PMC3409338 22855643

[169] Motallaei M, Ramezani-Jolfaie N, Mohammadi M, Shams‐Rad S, Jahanlou AS, Salehi‐Abargouei A (2021) Effects of orange juice intake on cardiovascular risk factors: a systematic review and meta‐analysis of randomized controlled clinical trials. Phytother Res 35(10):10. 10.1002/ptr.7173 34060162

[170] Ha V, Sievenpiper JL, De Souza RJ, Chiavaroli L, Wang DD, Cozma AI et al (2012) Effect of fructose on blood pressure: a systematic review and meta-analysis of controlled feeding trials. Hypertension 59(4):4. 10.1161/HYPERTENSIONAHA.111.182311 22331380

[171] Narain A, Kwok CS, Mamas MA (2016) Soft drinks and sweetened beverages and the risk of cardiovascular disease and mortality: a systematic review and meta-analysis. Int J Clin Pract 70(10):10. 10.1111/ijcp.12841 27456347

[172] Bechthold A, Boeing H, Schwedhelm C, Hoffmann G, Knüppel S, Iqbal K et al (2019) Food groups and risk of coronary heart disease, stroke and heart failure: a systematic review and dose-response meta-analysis of prospective studies. Crit Rev Food Sci Nutr. 10.1080/10408398.2017.1392288 29039970

[173] Mengoa MGR, Rangel BT, Garcia AAMN, Sugio CYC, Gomes ACG, Sugio CTC et al (2023) The challenge of dietary management in soft drink consumption and its oral and systemic repercussions. Nutr Health 29(3):3. 10.1177/02601060221136356 36349359

[174] Bhandari B, Zeng L, Grafenauer S, Schutte AE, Xu X (2024) Long-term consumption of 6 different beverages and cardiovascular disease–related mortality: a systematic review and meta-analysis of prospective cohort studies. Curr Developments Nutr 8(3):102095. 10.1016/j.cdnut.2024.102095 PMC10904171 38425440

[175] Kim J, Gunathilake M, Hoang T, Kim OY (2026) Associations of dietary intake with cardiovascular diseases, blood pressure, and lipid profile in the Korean population: an updated systematic review and meta-analysis. J Lipid Atheroscler 15(1):111. 10.12997/jla.2026.15.1.111 PMC12875075 41660529

[176] Jamali M, Anvarifard P, Hosseini B, Razaghi M, Khorraminezhad L, Alvandi E et al (2025) Sweetened beverages and cardiovascular outcomes: an umbrella review of mortality and health outcomes. Nutr J 24(1):173. 10.1186/s12937-025-01242-1 PMC12613916 41225521

[177] Ding P, Yue W, Wang X, Zhang Y, Liu Y, Guo X (2024) Effects of sugary drinks, coffee, tea and fruit juice on incidence rate, mortality and cardiovascular complications of type2 diabetes patients: a systematic review and meta-analysis. J Diabetes Metab Disord 23(1):1113–23. 10.1007/s40200-024-01396-5 PMC11196440 38932853

[178] Kim J, Hoang T, Bu SY, Kim JM, Choi JH, Park E et al (2020) Associations of dietary intake with cardiovascular disease, blood pressure, and lipid profile in the Korean population: a systematic review and meta-analysis. J Lipid Atheroscler 9(1):205–29. 10.12997/jla.2020.9.1.205 PMC7379076 32821732

[179] Sun T, Zhang Y, Ding L, Zhang Y, Li T, Li Q (2023) The relationship between major food sources of fructose and cardiovascular outcomes: a systematic review and dose-response meta-analysis of prospective studies. Adv Nutr 14(2):2. 10.1016/j.advnut.2022.12.002 PMC10229384 36803836

[180] Peters R, White D, Cleeland C, Scholey A (2020) Fuel for thought? a systematic review of neuroimaging studies into glucose enhancement of cognitive performance. Neuropsychol Rev 30(2):234–50. 10.1007/s11065-020-09431-x PMC7305087 32162177

[181] Del-Ponte B, Quinte GC, Cruz S, Grellert M, Santos IS (2019) Dietary patterns and attention deficit/hyperactivity disorder (ADHD): A systematic review and meta-analysis. J Affect Disord 252:160–173. 10.1016/j.jad.2019.04.061 30986731

[182] Cohen JFW, Gorski MT, Gruber SA, Kurdziel LBF, Rimm EB (2016) The effect of healthy dietary consumption on executive cognitive functioning in children and adolescents: a systematic review. Br J Nutr 116(6):989–1000. 10.1017/S0007114516002877 27487986

[183] Ishihara L, Brayne C (2005) A systematic review of nutritional risk factors of Parkinson’s disease. Nutr Res Rev 18(2):259–82. 10.1079/NRR2005108 19079910

[184] Van De Rest O, Van Der Zwaluw NL, De Groot LCPGM (2018) Effects of glucose and sucrose on mood: a systematic review of interventional studies. Nutr Rev 76(2):108–116. 10.1093/nutrit/nux065 29228399

[185] García CR, Piernas C, Martínez-Rodríguez A, Hernández-Morante JJ (2021) Effect of glucose and sucrose on cognition in healthy humans: a systematic review and meta-analysis of interventional studies. Nutr Rev 79(2):171–187. 10.1093/nutrit/nuaa036 32585003

[186] Kirvalidze M, Hodkinson A, Storman D, Fairchild TJ, Bała MM, Beridze G et al (2022) The role of glucose in cognition, risk of dementia, and related biomarkers in individuals without type 2 diabetes mellitus or the metabolic syndrome: a systematic review of observational studies. Neurosci Biobehav Rev 135:104551. 10.1016/j.neubiorev.2022.104551 35104494

[187] Sun Q, Yang Y, Wang X, Yang R, Li X (2022) The association between sugar-sweetened beverages and cognitive function in middle-aged and older people: a meta-analysis. J Prev Alzheimer’s Disease 9(2):323–330. 10.14283/jpad.2021.71 35543006

[188] Taylor ZB, Stevenson RJ, Ehrenfeld L, Francis HM (2021) The impact of saturated fat, added sugar and their combination on human hippocampal integrity and function: a systematic review and meta-analysis. Neurosci Biobehavioral Reviews 130:91–106. 10.1016/j.neubiorev.2021.08.008 34400179

[189] Liu H, Liu Y, Shi M, Zhou Y, Zhao Y, Xia Y (2022) Meta-analysis of sugar-sweetened beverage intake and the risk of cognitive disorders. J Affect Disord 313:177–185. 10.1016/j.jad.2022.06.048 35780967

[190] Hu D, Cheng L, Jiang W (2019) Sugar-sweetened beverages consumption and the risk of depression: a meta-analysis of observational studies. J Affect Disord 245:348–355. 10.1016/j.jad.2018.11.015 30419536

[191] Farsad-Naeimi A, Asjodi F, Omidian M, Askari M, Nouri M, Pizarro AB et al (2020) Sugar consumption, sugar sweetened beverages and Attention Deficit Hyperactivity Disorder: A systematic review and meta-analysis. Complement Ther Med 53:102512. 10.1016/j.ctim.2020.102512 33066852

[192] Mantantzis K, Schlaghecken F, Sünram-Lea SI, Maylor EA (2019) Sugar rush or sugar crash? A meta-analysis of carbohydrate effects on mood. Neurosci Biobehavioral Reviews 101:45–67. 10.1016/j.neubiorev.2019.03.016 30951762

[193] Khaled K, Abdulbaki N, Almilaji O, Casey C, Tsofliou F (2026) Sugar-sweetened beverage consumption and anxiety disorders in adolescents: a systematic review and meta‐analysis. J Hum Nutr Diet 39(1):e70217. 10.1111/jhn.70217 41664940

[194] Wang X, Wong ACW, Sheng Z, Wong SY, Yang X (2024) The relationship between dietary sugar consumption and anxiety disorders: A systematic review. Nutr Bull 49(4):429–443. 10.1111/nbu.12702 39138127

[195] Barma MD, Purohit BM, Priya H, Malhotra S, Bhadauria US, Duggal R (2026) Sweet misery: association of sugar consumption with anxiety and depression—a systematic review. Obes Rev 27(1):1–19. 10.1111/obr.70003 40715023

[196] Xiong J, Wang L, Huang H, Xiong S, Zhang S, Fu Q et al (2024) Association of sugar consumption with risk of depression and anxiety: a systematic review and meta-analysis. Front Nutr 11:1472612. 10.3389/fnut.2024.1472612 PMC11522855 39479195

[197] García CR, Piernas C, Martínez-Rodríguez A, Hernández-Morante JJ (2021) Effect of glucose and sucrose on cognition in healthy humans: a systematic review and meta-analysis of interventional studies. Nutr Rev 79(2):171–187. 10.1093/nutrit/nuaa036 PubMed PMID: 32585003 32585003

[198] Li H, Liang H, Yang H, Zhang X, Ding X, Zhang R et al (2022) Association between intake of sweetened beverages with all-cause and cause-specific mortality: a systematic review and meta-analysis. J Public Health 44(3):3. 10.1093/pubmed/fdab069 33837431

[199] Pan B, Ge L, Lai H, Wang Q, Wang Q, Zhang Q et al (2022) Association of soft drink and 100% fruit juice consumption with all-cause mortality, cardiovascular diseases mortality, and cancer mortality: A systematic review and dose-response meta-analysis of prospective cohort studies. Crit Rev Food Sci Nutr 62(32):32. 10.1080/10408398.2021.1937040 34121531

[200] Zhang YB, Jiang YW, Chen JX, Xia PF, Pan A (2021) Association of consumption of sugar-sweetened beverages or artificially sweetened beverages with mortality: a systematic review and dose–response meta-analysis of prospective cohort studies. Adv Nutr 12(2):2. 10.1093/advances/nmaa110 PMC8009739 33786594

[201] Beckett EL, Fayet-Moore F, Cassettari T, Starck C, Wright J, Blumfield M (2025) Health effects of drinking 100% juice: an umbrella review of systematic reviews with meta-analyses. Nutr Rev 83(2):e722–e735. 10.1093/nutrit/nuae036 PMC11723140 38679915

[202] Lane MM, Travica N, Gamage E, Marshall S, Trakman GL, Young C et al (2024) Sugar-sweetened beverages and adverse human health outcomes: an umbrella review of meta-analyses of observational studies. Annu Rev Nutr 44(1):383–404. 10.1146/annurev-nutr-062322-020650 39207876

[203] Boushey C, Ard J, Bazzano L, Heymsfield S, Mayer-Davis E, Sabaté J et al Dietary patterns and all-cause mortality: a systematic review [Internet]. Alexandria (VA): USDA Nutrition Evidence Systematic Review; 2020 [cited 2024 Nov 15]. (USDA Nutrition Evidence Systematic Reviews). Available from: http://www.ncbi.nlm.nih.gov/books/NBK578477/ PubMed PMID: 35258870 35258870

[204] Taneri PE, Wehrli F, Roa-Díaz ZM, Itodo OA, Salvador D, Raeisi-Dehkordi H et al (2022) Association between ultra-processed food intake and all-cause mortality: a systematic review and meta-analysis. Am J Epidemiol. 10.1093/aje/kwac039 35231930

[205] Onni AT, Balakrishna R, Perillo M, Amato M, Javadi Arjmand E, Thomassen LM et al (2025) Umbrella review of systematic reviews and meta-analyses on consumption of different food groups and risk of all-cause mortality. Adv Nutr 16(4):100393. 10.1016/j.advnut.2025.100393 PMC11931306 39956388

[206] Federici C, Detzel P, Petracca F, Dainelli L, Fattore G (2019) The impact of food reformulation on nutrient intakes and health, a systematic review of modelling studies. BMC Nutr 5(1):1. 10.1186/s40795-018-0263-6 PMC7050750 32154802

[207] Rafie N, Golpour Hamedani S, Barak F, Safavi SM, Miraghajani M (2017) Dietary patterns, food groups and telomere length: a systematic review of current studies. Eur J Clin Nutr 71(2):151–158. 10.1038/ejcn.2016.149 27530475

[208] Valera-Gran D, Prieto-Botella D, Hurtado-Pomares M, Baladia E, Petermann-Rocha F, Sánchez-Pérez A et al (2022) The impact of foods, nutrients, or dietary patterns on telomere length in childhood and adolescence: a systematic review. Nutrients 14(19):3885. 10.3390/nu14193885 PMC9570627 36235538

[209] Ayoub-Charette S, Chiavaroli L, Liu Q, Khan TA, Zurbau A, Au-Yeung F et al (2021) Different food sources of fructose-containing sugars and fasting blood uric acid levels: a systematic review and meta-analysis of controlled feeding trials. J Nutr 151(8):2409–2421. 10.1093/jn/nxab144 PMC8349131 34087940

[210] Wang DD, Sievenpiper JL, De Souza RJ, Cozma AI, Chiavaroli L, Ha V et al (2014) Effect of fructose on postprandial triglycerides: a systematic review and meta-analysis of controlled feeding trials. Atherosclerosis 232(1):125–33. 10.1016/j.atherosclerosis.2013.10.019 24401226

[211] Schwingshackl L, Hoffmann G, Iqbal K, Schwedhelm C, Boeing H (2018) Food groups and intermediate disease markers: a systematic review and network meta-analysis of randomized trials. Am J Clin Nutr 108(3):576–586. 10.1093/ajcn/nqy151 PMC6134288 30535089

[212] Livesey G, Taylor R, Livesey HF, Buyken AE, Jenkins DJA, Augustin LSA et al (2019) Dietary glycemic index and load and the risk of type 2 diabetes: a systematic review and updated meta-analyses of prospective cohort studies. Nutrients 11(6):6. 10.3390/nu11061280 PMC6627334 31195724

[213] Nikniaz L, Abbasalizad-Farhangi M, Vajdi M, Nikniaz Z (2021) The association between sugars sweetened beverages (SSBs) and lipid profile among children and youth: a systematic review and dose‐response meta‐analysis of cross‐sectional studies. Pediatr Obes 16(7):e12782. 10.1111/ijpo.12782 33629539

[214] Evans RA, Frese M, Romero J, Cunningham JH, Mills KE (2017) Chronic fructose substitution for glucose or sucrose in food or beverages has little effect on fasting blood glucose, insulin, or triglycerides: a systematic review and meta-analysis. Am J Clin Nutr 106(2):519–529. 10.3945/ajcn.116.145169 28592603

[215] Zhang YH, An T, Zhang RC, Zhou Q, Huang Y, Zhang J (2013) Very high fructose intake increases serum LDL-cholesterol and total cholesterol: a meta-analysis of controlled feeding trials. J Nutr 143(9):1391–1398. 10.3945/jn.113.175323 23825185

[216] Gibson SA (2007) Dietary sugars intake and micronutrient adequacy: a systematic review of the evidence. Nutr Res Rev 20(2):121–131. 10.1017/S0954422407797846 19079865

[217] Macedo RCO, Vieira AF, Moritz CEJ, Reischak-Oliveira A (2018) Effects of fructose consumption on postprandial TAG: an update on systematic reviews with meta-analysis. Br J Nutr 120(4):364–372. 10.1017/S0007114518001538 29962368

[218] Chiavaroli L, De Souza RJ, Ha V, Cozma AI, Mirrahimi A, Wang DD et al (2015) Effect of fructose on established lipid targets: a systematic review and meta-analysis of controlled feeding trials. JAHA 4(9):e001700. 10.1161/JAHA.114.001700 PMC4599489 26358358

[219] Livesey G, Taylor R, Hulshof T, Howlett J (2008) Glycemic response and health–a systematic review and meta-analysis: relations between dietary glycemic properties and health outcomes. Am J Clin Nutr 87(1):258S-268S. 10.1093/ajcn/87.1.258S 18175766

[220] Händel MN, Heitmann BL, Abrahamsen B (2015) Nutrient and food intakes in early life and risk of childhood fractures: a systematic review and meta-analysis. Am J Clin Nutr 102(5):1182–1195. 10.3945/ajcn.115.108456 26447151

[221] Ahn H, Park YK (2021) Sugar-sweetened beverage consumption and bone health: a systematic review and meta-analysis. Nutr J 20(1):41. 10.1186/s12937-021-00698-1 PMC8101184 33952276

[222] Boushey C, Ard J, Bazzano L, Heymsfield S, Mayer-Davis E, Sabaté J et al Dietary Patterns and Bone Health: A systematic review [Internet]. U.S. department of agriculture, food and nutrition service, center for nutrition policy and promotion, nutrition evidence systematic review; 2020 Jul [cited 2025 May 10]. Available from: https://nesr.usda.gov/2020-dietary-guidelines-advisory-committee-systematic-reviews/dietary-patterns-subcommittee/dietary-patterns-bone-health 35129903

[223] Cara KC, Beauchesne AR, Wallace TC, Chung M (2022) Effects of 100% orange juice on markers of inflammation and oxidation in healthy and at-risk adult populations: a scoping review, systematic review, and meta-analysis. Adv Nutr 13(1):116–137. 10.1093/advances/nmab101 PMC8803484 34634114

[224] Qi X, Chiavaroli L, Lee D, Ayoub-Charette S, Khan TA, Au-Yeung F et al (2022) Effect of important food sources of fructose-containing sugars on inflammatory biomarkers: a systematic review and meta-analysis of controlled feeding trials. Nutrients 14(19):3986. 10.3390/nu14193986 PMC9572084 36235639

[225] Della Corte KW, Perrar I, Penczynski KJ, Schwingshackl L, Herder C, Buyken AE (2018) Effect of dietary sugar intake on biomarkers of subclinical inflammation: a systematic review and meta-analysis of intervention studies. Nutrients 10(5):5. 10.3390/nu10050606 PMC5986486 29757229

[226] Alwis US, Haddad R, Monaghan TF, Abrams P, Dmochowski R, Bower W et al (2020) Impact of food and drinks on urine production: a systematic review. Int J Clin Pract 74(9):9. 10.1111/ijcp.13539 32441853

[227] Cheungpasitporn W, Thongprayoon C, O’Corragain OA, Edmonds PJ, Kittanamongkolchai W, Erickson SB (2014) Associations of sugar-sweetened and artificially sweetened soda with chronic kidney disease: a systematic review and meta-analysis. Nephrology 19(12):12. 10.1111/nep.12343 25251417

[228] Lo WC, Ou SH, Chou CL, Chen JS, Wu MY, Wu MS (2021) Sugar- and artificially-sweetened beverages and the risks of chronic kidney disease: a systematic review and dose–response meta-analysis. J Nephrol 34(6):6. 10.1007/s40620-020-00957-0 33502726

[229] Shahdadian F, Boozari B, Saneei P (2023) Association between short sleep duration and intake of sugar and sugar-sweetened beverages: a systematic review and meta-analysis of observational studies. Sleep Health 9(2):2. 10.1016/j.sleh.2022.07.006 36424247

[230] Al-Zalabani AH, Noor Elahi I, Katib A, Alamri AG, Halawani A, Alsindi NM et al (2019) Association between soft drinks consumption and asthma: a systematic review and meta-analysis. BMJ Open 9(10):e029046. 10.1136/bmjopen-2019-029046 PMC6797295 31615794

[231] Keshavarz F, Zeinalabedini M, Ebrahimpour-Koujan S, Azadbakht L (2024) A systematic review and meta-analysis of the association of all types of beverages high in fructose with asthma in children and adolescents. BMC Nutr 10(1):123. 10.1186/s40795-024-00930-1 PMC11409540 39294754

[232] Moores CJ, Kelly SAM, Moynihan PJ (2022) Systematic review of the effect on caries of sugars intake: ten-year update. J Dent Res 101(9):9. 10.1177/00220345221082918 35302414

[233] Li Y (2011) Controlling sugar consumption still has a role to play in the prevention of dental caries. J Evid Based Dent Pract 11(1):1. 10.1016/j.jebdp.2010.11.012 21420004

[234] Burt BA, Pai S (2001) Sugar consumption and caries risk: a systematic review. J Dent Educ 65(10):10. 10.1002/j.0022-0337.2001.65.10.tb03444.x 11699972

[235] Echeverria MS, Mathias FB, Schuch HS, Cenci MS, Correa MB, Huysmans MC et al (2025) Sugar consumption and early childhood caries: a systematic review and meta-analysis of cohort studies. Braz oral res 39:e122. 10.1590/1807-3107bor-2025.vol39.122 PMC12628725 41259577

[236] Akinsolu FT, Gbaja-Biamila TA, Salako AO, Adewole IE, Olagunju MT, Abodunrin OR et al (2024) A systematic review and meta-analysis of the association between sugar intake and dental caries in Nigeria. BMC Oral Health 24(1):1532. 10.1186/s12903-024-05309-z PMC11662489 39709412

[237] Sandy LPA, Helmyati S, Amalia R (2024) Nutritional factors associated with early childhood caries: A systematic review and meta-analysis. Saudi Dent J 36(3):413–419. 10.1016/j.sdentj.2023.12.001 PMC10960096 38525179

[238] Moynihan PJ, Kelly S, a. M (2014) Effect on caries of restricting sugars intake: systematic review to inform WHO guidelines. J Dent Res 93(1):8–18 doi:10.1177/0022034513508954 PubMed PMID: 24323509; PubMed Central PMCID: PMC3872848 10.1177/0022034513508954 PMC3872848 24323509

